# Supplementary material for: Long-term risk of inflammatory bowel disease after endoscopic biopsy with normal mucosa: A population-based, sibling-controlled cohort study in Sweden
Source: PLoS Med. 2023 Feb 23;20(2):e1004185. doi: 10.1371/journal.pmed.1004185 (PMC9949679; doi:10.1371/journal.pmed.1004185)
Supplement: S1 Appendix — Fig A. Standardized cumulative incidence and 95% CI of inflammatory bowel disease in individuals with a GI biopsy result of normal mucosa (pink) and their matched population references (blue), stratified by sex, age at index date, or calendar period at index date. Table A. ICD codes and SNOMED codes defining IBD. Table B. Definitions of endoscopy, colectomy, and proctocolectomy. Table C. ICD codes assigned for phenotypes of IBD. Table D. Anatomical Therapeutic Chemical codes representing IBD treatment. Table E. Incidence rate of IBD in individuals with a GI biopsy result of normal mucosa and their matched population references. Table F. Incidence rate of IBD in individuals with a GI biopsy result of normal mucosa and their unexposed full siblings. Table G. Cumulative incidence and 95% CI of IBD during follow-up in individuals with a GI biopsy result of normal mucosa, compared with their matched population references. Table H. Cumulative incidence and 95% CI of IBD during follow-up in individuals with a GI biopsy result of normal mucosa, compared with their unexposed full siblings. Table I. Subgroup analyses of IBD during follow-up in individuals with a lower GI biopsy result of normal mucosa, compared with their matched population references. Table J. Subgroup analyses of IBD during follow-up in individuals with a lower GI biopsy result of normal mucosa, compared with their unexposed full siblings. Table K. Associations between lower GI biopsy result of normal mucosa and risk of IBD phenotypes, compared with their matched population references. Table L. Characteristics of individuals with an upper GI biopsy of normal mucosa and their matched population references and unexposed full siblings. Table M. Sensitivity analyses of IBD during follow-up in individuals with a lower GI biopsy result of normal mucosa, compared with their matched population references. (DOCX) [file pmed.1004185.s002.docx]

**Long-term risk of inflammatory bowel disease after endoscopic biopsy with normal mucosa: A population-based, sibling-controlled cohort study in Sweden**

Sun J et al.

Content:

Fig A: Standardized cumulative incidence and 95% confidence interval (CI) of inflammatory bowel disease in individuals with a gastrointestinal (GI) biopsy result of normal mucosa (pink) and their matched population references (blue), stratified by sex, age at index date, or calendar period at index date.

Table A. International Classification of Disease (ICD) codes and SNOMED codes defining inflammatory bowel diseases (IBD).

Table B. Definitions of endoscopy, colectomy, and proctocolectomy.

Table C. ICD codes assigned for phenotypes of IBD.

Table D. Anatomical Therapeutic Chemical (ATC) codes representing IBD treatment.

Table E. Incidence rate of IBD in individuals with a GI biopsy result of normal mucosa and their matched population references.

Table F. Incidence rate of IBD in individuals with a GI biopsy result of normal mucosa and their unexposed full siblings.

Table G. Cumulative incidence and 95%CI of IBD during follow-up in individuals with a GI biopsy result of normal mucosa, compared with their matched population references.

Table H. Cumulative incidence and 95%CI of IBD during follow-up in individuals with a GI biopsy result of normal mucosa, compared with their unexposed full siblings.

Table I. Subgroup analyses of IBD during follow-up in individuals with a lower GI biopsy result of normal mucosa, compared with their matched population references.

Table J. Subgroup analyses of IBD during follow-up in individuals with a lower GI biopsy result of normal mucosa, compared with their unexposed full siblings.

Table K. Associations between lower GI biopsy result of normal mucosa and risk of IBD phenotypes, compared with their matched population references.

Table L. Characteristics of individuals with an upper GI biopsy of normal mucosa and their matched population references and unexposed full siblings.

Table M. Sensitivity analyses of IBD during follow-up in individuals with a lower GI biopsy result of normal mucosa, compared with their matched population references.


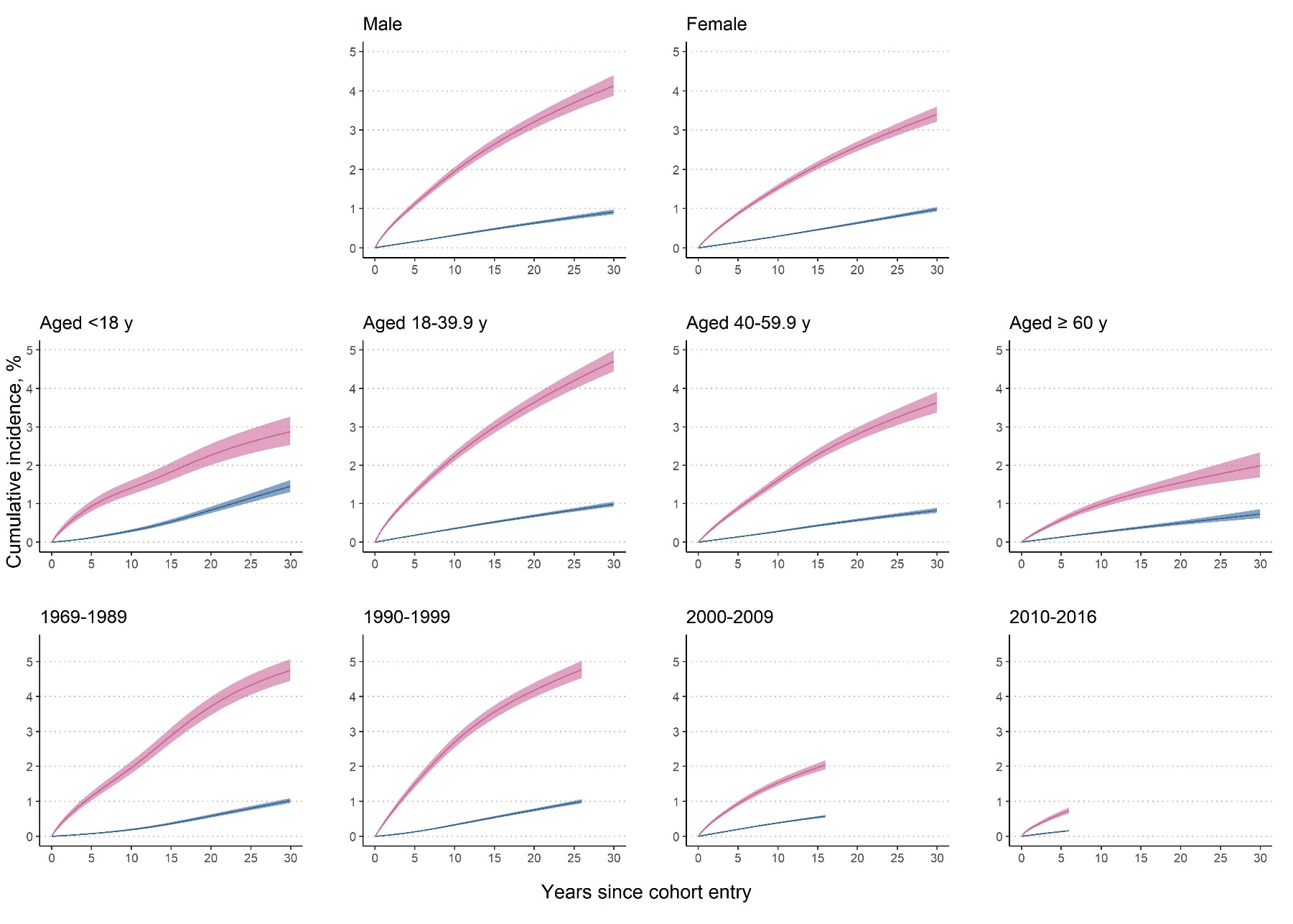


Fig A. Standardized cumulative incidence and 95% confidence interval of inflammatory bowel disease in individuals with a gastrointestinal (GI) biopsy result of normal mucosa (pink) and their matched population references (blue), stratified by sex, age at index date, or calendar period at index date. Date of cohort entry was defined as 6 months after the index date

| Table A. International Classification of Disease (ICD) codes and SNOMED codes defining inflammatory bowel diseases (IBD) ^a^ | | | | | |
| --- | --- | --- | --- | --- | --- |
| IBD subtypes | ICD-7 (1964-1968) | ICD-8 (1969-1986) | ICD-9 (1987-1996) | ICD-10 (1997-) | SNOMED codes ^b^ |
| Ulcerative colitis (UC) | 572,20; 572,21; 578,03 | 563,1; 563,10; 569,02; 569,04 | 556 | K51 | D6255 or M41, M42, M43, M44, M463, or M47 |
| Crohn's disease (CD) | 572,00; 572,09 | 563,00 | 555 | K50 | D6216 or M41, M42, M43, M44, M463, or M47 |
| IBD unclassified (IBD-U) | UC + CD | UC + CD or 563; 563,0; 563,9; 563,98; 563,99 | UC + CD | UC + CD or K52.3 | D6214 or M41, M42, M43, M44, M463, or M47 |
| ^a^ Subtypes of IBD were defined according to the first two diagnostic codes only, therefore no information after start of follow-up contributed to such definition; and the IBD subtype was only determined by the ICD code if one individual had one ICD code for IBD and one M code. ≥1 ICD code for IBD plus a relevant biopsy code has a positive predictive value of 95% [1,2]. In a recent paper [3], we report that 18% of incident IBD patients in the Swedish patient register during 2002-2014 were classified as another IBD subtype at some point during follow-up. | | | | | |
| ^b^ SNOMED codes starting with M (inflammation suggestive of IBD, but not a specified subtype) were required to be accompanied by a topographic code of T67 or T68 (colon); for example, M41 refers to all codes starting with M41; D codes are diagnostic codes but listed under morphology in pathology registers; D6255 for example is the diagnostic code for UC. | | | | | |

| Table B. Definitions of endoscopy, colectomy, and proctocolectomy | | |
| --- | --- | --- |
| Procedures | Classification of procedures | Definition code |
| Endoscopy | Esophagogastroduodenoscopy | 2861, 2880, 2881, 4480, 4483, 4486, 4487, 4488, 4489, 4490, 9021, 4686, 4687, 9003, 9004, 9021, UJC, UJD, UJF02, UJF05 |
|  | Colonoscopy | 9011, 9023, 4688, 4689, UJF32, UJF35 |
|  | Sigmoidoscopy | 9012, 4685, UJF42, UJF45 |
|  | Colonoscopy or sigmoidoscopy | 4674, 4684 |
| Colectomy | Sixth revision | 4650, 4651 |
|  | Seventh revision | JFH10, JFH11, JFH96, JFH00, JFH01, JFC40, JFC41, JFG29, JFG26 |
| Proctocolectomy | Sixth revision | 4652, 4653, 4654 |
|  | Seventh revision | JFH30, JFH33, JGB50, JGB60, JFH40, JFH20 |
| Gastrointestinal disease | ICD-7 (1964-1968) | 530-587 |
|  | ICD-8 (1969-1986) | 520-579 |
|  | ICD-9 (1987-1996) | 520-579 |
|  | ICD-10 (1997-) | K00-K99 |

| Table C. ICD codes assigned for phenotypes of IBD | |
| --- | --- |
| Montreal classification | Diagnostic codes |
| Crohn’s disease location ^a^ |  |
| Ileal (L1) | K50.0 |
| Colonic (L2) | K50.1 |
| Ileocolonic or location not defined (L3/LX) | K50.8, K50.9 |
| Ulcerative colitis extent ^a^ |  |
| Proctitis (E1) | K51.2 |
| Left-sided colitis (E2) | K51.3; K51.5 |
| Extensive colitis (E3) | K51.0 |
| Extent not defined (EX) | K51.4; K51.8; K51.9 |
| ^a^ Validated definitions and diagnostic codes [4] used to define Crohn’s disease and ulcerative colitis according to the Montreal classification since the start of use of the ICD-10 in Sweden (1997-). All codes are captured in the Swedish National Patient Register (prospectively recorded in routine clinical practice). | |

| Table D. Anatomical Therapeutic Chemical(ATC) codes representing IBD treatment | | |
| --- | --- | --- |
| Drug group | Substance | ATC code |
| Immunomodulators | Azathioprine | L04AX01 |
|  | Mercaptopurine | L01BB02 |
|  | Methotrexate | L04AX03/L01BA01 |
| Anti-TNF treatment | Infliximab | L04AB02 (L04AA12 before 2008) |
|  | Adalimumab | L04AB04 (L04AA17 before 2008) |
|  | Golimumab | L04AB06 |
|  | Vedolizumab | L04AA33 |
| Systemic corticosteroids | Betamethasone | H02AB01 |
|  | Dexamethasone | H02AB02 |
|  | Methylprednisolone | H02AB04 |
|  | Prednisolone | H02AB06 |
|  | Prednisone | H02AB07 |
|  | Hydrocortisone | H02AB09 |
|  | Cortisone | H02AB10 |
| Systemic aminosalicylates (5-ASA) | Sulfasalazine | A07EC01 |
|  | Mesalazine | A07EC02 |
|  | Olsalazine | A07EC03 |
|  | Balsalazide | A07EC04 |
| Rectal aminosalicylates (5-ASA) | Mesalazine | A07EC02 |
| Corticosteroids acting locally | Hydrocortisone | A07EA02 |
|  | Budesonide | A07EA06 |

| Table E. Incidence rate of IBD in individuals with a GI biopsy result of normal mucosa and their matched population references | | | | | | | |
| --- | --- | --- | --- | --- | --- | --- | --- |
|  | No. of events, n (%) | |  | Incidence rate (95% CI), per 10,000 Pys | |  | Incidence rate difference (95% CI), per 10,000 Pys |
| Biopsy location and outcomes | Normal mucosa | References |  | Normal mucosa | References |  |  |
| Lower GI and IBD | 4853 (2.4) | 4100 (0.4) |  | 20.39 (19.82-20.98) | 3.39 (3.28-3.49) |  | 17.01 (16.43-17.59) |
| Lower GI and UC | 2339 (1.2) | 2206 (0.2) |  | 9.83 (9.44-10.24) | 1.82 (1.75-1.90) |  | 8.01 (7.60-8.41) |
| Lower GI and CD | 1789 (0.9) | 1142 (0.1) |  | 7.52 (7.17-7.87) | 0.94 (0.89-1.00) |  | 6.57 (6.22-6.93) |
| Upper GI and CD | 1048 (0.4) | 1532 (0.1) |  | 3.37 (3.17-3.58) | 0.97 (0.93-1.02) |  | 2.39 (2.18-2.60) |
| CD: Crohn's disease; CI: confidence interval; IBD: inflammatory bowel disease; UC: ulcerative colitis | | | | | | | |

| Table F. Incidence rate of IBD in individuals with a GI biopsy result of normal mucosa and their unexposed full siblings | | | | | | | |
| --- | --- | --- | --- | --- | --- | --- | --- |
|  | No. of events, n (%) | |  | Incidence rate (95% CI), per 10,000 Pys | |  | Incidence rate difference (95% CI), per 10,000 Pys |
| Biopsy location and outcomes | Normal mucosa | Unexposed full siblings |  | Normal mucosa | Unexposed full siblings |  |  |
| Lower GI and IBD | 3216 (2.7) | 1625 (0.7) |  | 21.53 (20.79-22.28) | 5.65 (5.38-5.93) |  | 15.87 (15.08-16.67) |
| Lower GI and UC | 1573 (1.3) | 817 (0.4) |  | 10.53 (10.02-11.06) | 2.84 (2.65-3.04) |  | 7.69 (7.13-8.24) |
| Lower GI and CD | 1177 (1.0) | 509 (0.2) |  | 7.88 (7.43-8.34) | 1.77 (1.62-1.93) |  | 6.11 (5.63-6.58) |
| Upper GI and CD | 687 (0.5) | 483 (0.2) |  | 3.66 (3.39-3.94) | 1.33 (1.21-1.45) |  | 2.33 (2.03-2.63) |
| CD: Crohn's disease; CI: confidence interval; IBD: inflammatory bowel disease; UC: ulcerative colitis | | | | | | | |

| Table G. Cumulative incidence and 95%CI of IBD during follow-up in individuals with a GI biopsy result of normal mucosa, compared with their matched population references | | | | | | |
| --- | --- | --- | --- | --- | --- | --- |
| Biopsy location and outcomes | Years since cohort entry | | | | | |
|  | 6 months | 1 year | 5 years | 10 years | 20 years | 30 years |
| Lower GI and IBD |  |  |  |  |  |  |
| Reference | 0.02 (0.01-0.02) | 0.03 (0.03-0.03) | 0.15 (0.14-0.16) | 0.31 (0.29-0.32) | 0.63 (0.61-0.66) | 0.96 (0.92-1.00) |
| Normal mucosa | 0.14 (0.13-0.16) | 0.26 (0.24-0.28) | 0.97 (0.93-1.02) | 1.69 (1.63-1.76) | 2.82 (2.72-2.92) | 3.67 (3.52-3.83) |
| Lower GI and UC |  |  |  |  |  |  |
| Reference | 0.01 (0.01-0.01) | 0.02 (0.02-0.02) | 0.08 (0.08-0.09) | 0.16 (0.15-0.17) | 0.32 (0.31-0.34) | 0.47 (0.44-0.50) |
| Normal mucosa | 0.06 (0.05-0.07) | 0.11 (0.10-0.13) | 0.47 (0.44-0.50) | 0.84 (0.80-0.89) | 1.43 (1.36-1.51) | 1.79 (1.69-1.89) |
| Lower GI and CD |  |  |  |  |  |  |
| Reference | 0.00 (0.00-0.01) | 0.01 (0.01-0.01) | 0.04 (0.04-0.05) | 0.09 (0.08-0.10) | 0.17 (0.16-0.19) | 0.26 (0.24-0.28) |
| Normal mucosa | 0.07 (0.06-0.08) | 0.12 (0.10-0.13) | 0.37 (0.34-0.39) | 0.61 (0.57-0.64) | 0.93 (0.88-0.99) | 1.20 (1.12-1.28) |
| Upper GI and CD |  |  |  |  |  |  |
| Reference | 0.00 (0.00-0.01) | 0.01 (0.01-0.01) | 0.05 (0.05-0.05) | 0.10 (0.09-0.10) | 0.19 (0.18-0.20) | 0.27 (0.25-0.29) |
| Normal mucosa | 0.03 (0.02-0.03) | 0.05 (0.04-0.06) | 0.16 (0.14-0.17) | 0.27 (0.25-0.29) | 0.48 (0.45-0.52) | 0.69 (0.62-0.75) |
| CD: Crohn's disease; CI: confidence interval; GI: gastrointestinal; IBD: inflammatory bowel disease; UC: ulcerative colitis. Cumulative incidence difference was estimated from the flexible parametric survival model, allowing normal mucosa to vary over time. All models were conditioned on matching set (birth year, sex, county of residence, and calendar period) and further adjusted for country of birth, educational attainment, number of healthcare visits, Charlson comorbidity index, and history of GI diseases. | | | | | | |

| Table H. Cumulative incidence and 95%CI of IBD during follow-up in individuals with a GI biopsy result of normal mucosa, compared with their unexposed full siblings | | | | | | |
| --- | --- | --- | --- | --- | --- | --- |
| Biopsy location and outcomes | Years since cohort entry | | | | | |
|  | 6 months | 1 year | 5 years | 10 years | 20 years | 30 years |
| Lower GI and IBD |  |  |  |  |  |  |
| Siblings | 0.04 (0.03-0.04) | 0.07 (0.06-0.08) | 0.32 (0.29-0.34) | 0.61 (0.57-0.65) | 1.13 (1.07-1.19) | 1.56 (1.46-1.66) |
| Normal mucosa | 0.16 (0.14-0.18) | 0.30 (0.27-0.32) | 1.13 (1.07-1.18) | 1.98 (1.90-2.06) | 3.28 (3.15-3.41) | 4.22 (4.02-4.43) |
| Lower GI and UC |  |  |  |  |  |  |
| Siblings | 0.02 (0.01-0.02) | 0.04 (0.03-0.04) | 0.15 (0.14-0.17) | 0.29 (0.27-0.32) | 0.54 (0.50-0.58) | 0.71 (0.65-0.78) |
| Normal mucosa | 0.07 (0.06-0.08) | 0.13 (0.11-0.15) | 0.53 (0.49-0.57) | 0.99 (0.93-1.05) | 1.66 (1.56-1.75) | 2.05 (1.92-2.19) |
| Lower GI and CD |  |  |  |  |  |  |
| Siblings | 0.01 (0.01-0.02) | 0.02 (0.02-0.03) | 0.11 (0.09-0.12) | 0.20 (0.18-0.22) | 0.35 (0.32-0.38) | 0.48 (0.43-0.53) |
| Normal mucosa | 0.08 (0.06-0.09) | 0.14 (0.12-0.15) | 0.45 (0.42-0.49) | 0.73 (0.68-0.78) | 1.11 (1.04-1.19) | 1.42 (1.31-1.53) |
| Upper GI and CD |  |  |  |  |  |  |
| Siblings | 0.01 (0.00-0.01) | 0.01 (0.01-0.02) | 0.08 (0.07-0.09) | 0.15 (0.13-0.17) | 0.28 (0.25-0.30) | 0.38 (0.33-0.43) |
| Normal mucosa | 0.03 (0.03-0.04) | 0.06 (0.05-0.07) | 0.20 (0.18-0.22) | 0.34 (0.31-0.37) | 0.59 (0.54-0.64) | 0.81 (0.72-0.91) |
| CD: Crohn's disease; CI: confidence interval; GI: gastrointestinal; IBD: inflammatory bowel disease; UC: ulcerative colitis. Cumulative incidence difference was estimated from the flexible parametric survival model, allowing normal mucosa to vary over time. All models were conditioned on family identifier and adjusted for birth year, sex, county of residence, calendar period, country of birth, educational attainment, number of healthcare visits, Charlson comorbidity index, and history of GI diseases | | | | | | |

| Table I. Subgroup analyses of IBD during follow-up in individuals with a lower GI biopsy result of normal mucosa, compared with their matched population references | | | | | | | | | | |
| --- | --- | --- | --- | --- | --- | --- | --- | --- | --- | --- |
| Subgroup ^a^ | No. of events | | Average HR (95%CI) |  | Years since cohort entry, HR (95%CI) | | | | | |
|  | Normal mucosa | References |  |  | 6 months | 1 year | 5 years | 10 years | 20 years | 30 years |
| Sex |  |  |  |  |  |  |  |  |  |  |
| Male | 2145 | 1583 | 6.24 (5.77-6.76) |  | 8.68 (7.32-10.30) | 7.92 (6.83-9.19) | 5.79 (5.22-6.43) | 4.78 (4.36-5.24) | 3.66 (3.18-4.21) | 3.14 (2.54-3.88) |
| Female | 2708 | 2517 | 5.11 (4.78-5.46) |  | 7.58 (6.54-8.78) | 6.78 (5.96-7.70) | 4.94 (4.54-5.38) | 3.89 (3.58-4.22) | 2.68 (2.40-3.00) | 2.16 (1.82-2.57) |
| Age at index date |  |  |  |  |  |  |  |  |  |  |
| <18 y | 426 | 623 | 3.29 (2.84-3.83) |  | 15.79 (10.77-23.14) | 12.49 (8.98-17.36) | 4.16 (3.41-5.06) | 1.96 (1.54-2.49) | 1.28 (1.03-1.60) | 0.80 (0.51-1.27) |
| 18-39.9 y | 2340 | 1737 | 6.38 (5.91-6.88) |  | 8.97 (7.60-10.58) | 7.80 (6.75-9.02) | 5.92 (5.35-6.54) | 5.01 (4.57-5.49) | 3.94 (3.48-4.46) | 3.44 (2.86-4.14) |
| 40-59.9 y | 1556 | 1189 | 5.72 (5.22-6.27) |  | 7.37 (5.99-9.06) | 6.98 (5.83-8.36) | 5.63 (5.02-6.32) | 4.82 (4.32-5.37) | 3.66 (3.11-4.32) | 3.10 (2.38-4.04) |
| ≥60 y | 531 | 551 | 4.06 (3.52-4.67) |  | 4.97 (3.73-6.62) | 4.76 (3.74-6.05) | 3.80 (3.14-4.61) | 2.82 (2.34-3.39) | 2.06 (1.42-2.99) | 1.77 (1.11-2.83) |
| Calendar period at index date | |  |  |  |  |  |  |  |  |  |
| 1969-1989 | 1086 | 901 | 5.76 (5.21-6.36) |  | 24.21 (16.83-34.83) | 20.10 (14.82-27.27) | 9.36 (7.91-11.07) | 6.18 (5.39-7.08) | 3.40 (2.99-3.85) | 1.76 (1.38-2.25) |
| 1990-1999 | 1891 | 1414 | 6.12 (5.67-6.61) |  | 17.81 (14.02-22.62) | 15.55 (12.72-19.01) | 7.85 (7.08-8.70) | 4.89 (4.38-5.47) | 2.73 (2.33-3.20) | - |
| 2000-2009 | 1471 | 1457 | 3.98 (3.66-4.32) |  | 6.02 (5.01-7.23) | 5.35 (4.58-6.24) | 3.65 (3.23-4.12) | 2.95 (2.58-3.36) | - | - |
| 2010-2016 | 405 | 328 | 5.18 (4.38-6.13) |  | 6.57 (4.90-8.81) | 4.98 (3.95-6.29) | 3.95 (2.87-5.45) | - | - | - |
| Hazard ratio (HR) was estimated from the flexible parametric survival model. All models were conditioned on matching set (birth year, sex, county of residence, and calendar period) and further adjusted for country of birth, educational attainment, number of healthcare visits, Charlson comorbidity index, and history of GI diseases. | | | | | | | | | | |
| All *P* *_for interaction_* were <0.001. | | | | | | | | | | |

| Table J. Subgroup analyses of IBD during follow-up in individuals with a lower GI biopsy result of normal mucosa, compared with their unexposed full siblings | | | | | | | | | | |
| --- | --- | --- | --- | --- | --- | --- | --- | --- | --- | --- |
| Subgroup ^a^ | No. of events | | Average HR (95%CI) |  | Years since cohort entry, HR (95%CI) | | | | | |
|  | Normal mucosa | Unexposed full siblings |  |  | 6 months | 1 year | 5 years | 10 years | 20 years | 30 years |
| Sex |  |  |  |  |  |  |  |  |  |  |
| Male | 1419 | 832 | 3.64 (3.29-4.02) |  | 4.62 (3.70-5.77) | 4.43 (3.66-5.37) | 3.49 (3.04-4.01) | 3.02 (2.68-3.39) | 2.53 (2.12-3.03) | 2.31 (1.80-2.96) |
| Female | 1797 | 793 | 2.96 (2.69-3.26) |  | 3.67 (2.95-4.56) | 3.34 (2.76-4.04) | 2.81 (2.46-3.20) | 2.57 (2.29-2.88) | 2.26 (1.90-2.68) | 2.10 (1.62-2.71) |
| Age at index date |  |  |  |  |  |  |  |  |  |  |
| <18 y | 341 | 253 | 2.87 (2.33-3.55) |  | 8.14 (4.52-14.63) | 6.50 (3.94-10.72) | 3.09 (2.27-4.21) | 2.05 (1.51-2.79) | 1.44 (1.05-1.98) | 1.16 (0.73-1.84) |
| 18-39.9 y | 1766 | 749 | 3.60 (3.26-3.98) |  | 4.26 (3.44-5.28) | 4.09 (3.39-4.93) | 3.60 (3.14-4.13) | 3.17 (2.82-3.56) | 2.58 (2.19-3.03) | 2.29 (1.80-2.91) |
| 40-59.9 y | 961 | 495 | 3.38 (2.98-3.83) |  | 4.22 (3.15-5.64) | 3.93 (3.05-5.06) | 3.01 (2.56-3.54) | 2.99 (2.58-3.45) | 3.32 (2.58-4.27) | 3.50 (2.43-5.04) |
| ≥60 y | 148 | 128 | 1.86 (1.41-2.45) |  | 1.86 (1.06-3.26) | 2.10 (1.29-3.42) | 1.84 (1.29-2.63) | 1.93 (1.20-3.10) | 2.00 (0.88-4.59) | 2.03 (0.77-5.37) |
| Calendar period at index date | |  |  |  |  |  |  |  |  |  |
| 1969-1989 | 709 | 326 | 4.05 (3.52-4.65) |  | 22.19 (11.81-41.72) | 17.16 (10.38-28.35) | 6.92 (5.28-9.08) | 4.48 (3.66-5.49) | 2.70 (2.26-3.22) | 1.79 (1.29-2.47) |
| 1990-1999 | 1265 | 555 | 3.81 (3.43-4.23) |  | 6.52 (4.74-8.97) | 6.15 (4.69-8.08) | 4.82 (4.15-5.60) | 3.64 (3.12-4.25) | 2.06 (1.66-2.56) | - |
| 2000-2009 | 978 | 580 | 2.32 (2.07-2.60) |  | 3.76 (2.89-4.91) | 3.28 (2.63-4.09) | 1.96 (1.66-2.31) | 1.98 (1.64-2.38) | - | - |
| 2010-2016 | 264 | 164 | 2.07 (1.67-2.56) |  | 2.53 (1.79-3.58) | 1.93 (1.42-2.62) | 1.97 (1.28-3.03) | - | - | - |
| Hazard ratio (HR) was estimated from the flexible parametric survival model. All models were conditioned on family identifier and adjusted for birth year, sex, county of residence, calendar period, country of birth, educational attainment, number of healthcare visits, Charlson comorbidity index, and history of GI diseases.  All *P* *_for interaction_* were <0.001. | | | | | | | | | | |
|  |  |  |  |  |  |  |  |  |  |  |
|  |  |  |  |  |  |  |  |  |  |  |
|  |  |  |  |  |  |  |  |  |  |  |

| Table K. Associations between lower GI biopsy result of normal mucosa and risk of IBD phenotypes, compared with their matched population references | | | | | | | | | | |
| --- | --- | --- | --- | --- | --- | --- | --- | --- | --- | --- |
| Subgroup | No. of events | | Average HR (95%CI) |  | Years since cohort entry, HR (95%CI) | | | | | |
|  | Normal mucosa | References |  |  | 0.5 y | 1 y | 5 y | 10 y | 20 y | 30 y |
| Montreal Classification CD |  |  |  |  |  |  |  |  |  |  |
| L1, L3/LX (Ileal, ileocolonic or location not defined) | 1197 | 816 | 6.66 (5.97-7.44) |  | 13.11 (10.16-16.91) | 10.68 (8.58-13.30) | 5.58 (4.81-6.47) | 4.12 (3.59-4.72) | 3.49 (2.94-4.15) | 3.09 (2.41-3.96) |
| L2 (Colonic) | 202 | 208 | 4.09 (3.24-5.17) |  | 4.14 (2.51-6.82) | 4.45 (2.86-6.92) | 4.59 (3.37-6.25) | 3.94 (2.99-5.19) | 2.98 (1.95-4.55) | 2.58 (1.40-4.75) |
| Montreal Classification UC |  |  |  |  |  |  |  |  |  |  |
| E1/E2 (Proctitis, left-sided colitis) | 726 | 907 | 4.38 (3.89-4.94) |  | 5.40 (4.14-7.04) | 5.11 (4.06-6.44) | 4.88 (4.20-5.68) | 3.94 (3.42-4.55) | 2.27 (1.84-2.80) | 1.59 (1.12-2.25) |
| E3 (Extensive colitis) | 351 | 319 | 4.94 (4.09-5.96) |  | 4.93 (3.22-7.55) | 4.76 (3.32-6.83) | 5.29 (4.23-6.60) | 5.13 (4.07-6.47) | 3.77 (2.74-5.18) | 3.08 (1.81-5.24) |
| EX (Extent not defined) | 749 | 779 | 4.55 (4.02-5.15) |  | 4.40 (3.35-5.78) | 4.42 (3.49-5.61) | 5.02 (4.31-5.84) | 4.70 (4.05-5.46) | 3.44 (2.82-4.20) | 2.92 (2.16-3.95) |
| Hazard ratio (HR) was estimated from the flexible parametric survival model. All models were conditioned on matching set (birth year, sex, county of residence, and calendar period) and further adjusted for country of birth, educational attainment, number of healthcare visits, Charlson comorbidity index, and history of GI diseases. | | | | | | | | | | |

| Table L. Characteristics of individuals with an upper GI biopsy of normal mucosa and their matched population references and unexposed full siblings | | | | | | | |  |
| --- | --- | --- | --- | --- | --- | --- | --- | --- |
|  | Population matched cohort, No. (%) | | Standardized difference ^f^ |  | Sibling cohort, No. (%) | | | Standardized difference ^f^ |
| Characteristics | Normal mucosa (n=257,192) | References (n=1,268,897) |  |  | Normal mucosa (n=148,564) | | Unexposed full siblings (n=274,529) |  |
| Age at index date, years ^a^ |  |  |  |  |  | |  |  |
| Mean ± SD | 45.0 ± 20.3 | 44.8 ± 20.3 | 0.010 |  | 38.3 ± 17.0 | | 39.7 ± 17.2 | -0.080 |
| Median (IQR) | 44.7 (28.3-60.9) | 44.5 (28.2-60.6) |  |  | 37.7 (24.4-51.4) | | 40.0 (26.0-52.9) |  |
| <18 y | 19086 (7.4) | 95689 (7.5) | 0.041 |  | 14441 (9.7) | | 31347 (11.4) | 0.102 |
| 18-39.9 y | 90416 (35.2) | 448388 (35.3) |  |  | 65815 (44.3) | | 105731 (38.5) |  |
| 40-59.9 y | 80216 (31.2) | 397104 (31.3) |  |  | 50701 (34.1) | | 101380 (36.9) |  |
| ≥60 y | 67474 (26.2) | 327716 (25.8) |  |  | 17607 (11.9) | | 36071 (13.1) |  |
| Female | 162213 (63.1) | 801221 (63.1) | -0.001 |  | 94143 (63.4) | | 135177 (49.2) | 0.288 |
| Born in Nordic country | 231429 (90.0) | 1121514 (88.4) | -0.051 |  | 144857 (97.5) | | 265471 (96.7) | -0.048 |
| Calendar period at index date ^a^ |  |  | 0.000 |  |  | |  | 0.053 |
| 1969-1989 | 18099 (7.0) | 89070 (7.0) |  |  | 6862 (4.6) | | 15105 (5.5) |  |
| 1990-1999 | 74674 (29.0) | 368610 (29.1) |  |  | 39668 (26.7) | | 77250 (28.1) |  |
| 2000-2009 | 101725 (39.6) | 501575 (39.5) |  |  | 61769 (41.6) | | 112006 (40.8) |  |
| 2010-2016 | 62694 (24.4) | 309642 (24.4) |  |  | 40265 (27.1) | | 70168 (25.6) |  |
| Educational attainment |  |  | 0.026 |  |  | |  | 0.145 |
| 0-9 y | 60508 (23.5) | 299158 (23.6) |  |  | 28127 (18.9) | | 58614 (21.4) |  |
| 10-12 y | 97110 (37.8) | 467421 (36.8) |  |  | 61829 (41.6) | | 111078 (40.5) |  |
| ≥13 y | 62702 (24.4) | 312414 (24.6) |  |  | 40185 (27.1) | | 63910 (23.3) |  |
| Missing | 36872 (14.3) | 189904 (15.0) |  |  | 18423 (12.4) | | 40927 (14.9) |  |
| History before the index date ^a^ |  |  |  |  |  | |  |  |
| Comorbidity≥1 ^b^ | 59724 (23.2) | 181594 (14.3) | 0.230 |  | 27344 (18.4) | | 35013 (12.8) | 0.156 |
| GI disease | 113242 (44.0) | 190357 (15.0) | 0.671 |  | 63973 (43.1) | | 54189 (19.7) | 0.519 |
| Endoscopy ^c^ | 80554 (31.3) | 30167 (2.4) | 0.838 |  | 48426 (32.6) | | 12666 (4.6) | 0.771 |
| EGD | 80286 (31.2) | 27743 (2.2) | 0.845 |  | 48228 (32.5) | 10778 (3.9) | | 0.796 |
| Colonoscopy | 12924 (5.0) | 6643 (0.5) | 0.277 |  | 7105 (4.8) | 3716 (1.4) | | 0.200 |
| Sigmoidoscopy | 2020 (0.8) | 1882 (0.2) | 0.094 |  | 1041 (0.7) | | 992 (0.4) | 0.047 |
| Colectomy or proctocolectomy | 112 (0.0) | 226 (0.0) | 0.015 |  | 52 (0.0) | | 119 (0.0) | -0.004 |
| With healthcare visit ^d^ | 94233 (36.6) | 320852 (25.3) | 0.247 |  | 53849 (36.3) | | 72117 (26.3) | 0.216 |
| Colonoscopy during follow-up | 25822 (10.0) | 59742 (4.7) | 0.205 |  | 14853 (10.0) | | 15690 (5.7) | 0.160 |
| Follow-up time, years |  |  |  |  |  | |  |  |
| Median (IQR) | 11.1 (5.6-17.7) | 11.4 (5.9-18.0) | -0.037 |  | 11.7 (6.2-18.3) | | 12.4 (6.7-18.9) | -0.078 |
| 0.5-0.9 y ^e^ | 8079 (3.1) | 31136 (2.5) | 0.074 |  | 3012 (2.0) | | 3940 (1.4) | 0.118 |
| 1-4.9 y | 48322 (18.8) | 230461 (18.2) |  |  | 25717 (17.3) | | 43374 (15.8) |  |
| 5-9.9 y | 59642 (23.2) | 294143 (23.2) |  |  | 34127 (23.0) | | 61192 (22.3) |  |
| 10-19.9 y | 96188 (37.4) | 479679 (37.8) |  |  | 57400 (38.6) | | 107369 (39.1) |  |
| 20-29.9 y | 40785 (15.9) | 211441 (16.7) |  |  | 25731 (17.3) | | 52634 (19.2) |  |
| ≥30 y | 4176 (1.6) | 22037 (1.7) |  |  | 2577 (1.7) | | 6020 (2.2) |  |
| IQR, interquartile range; SD, standard deviation | | | | | | | |  |
| ^a^ Index date: date of first biopsy record for individuals with a gastrointestinal biopsy of normal mucosa, and date of selection for their matched population references or unexposed full siblings. | | | | | | | |  |
| ^b^ Measured by the Charlson comorbidity index.  ^c^ One individual may have multiple records of endoscopy before the index date. | | | | | | | |  |
| ^d^ Defined as the number of healthcare visits between 2 years and 6 months before the index date.  ^e^ Follow-up started 6 months after index date.  ^f^ Standardized difference defined as difference in means or proportions divided by standard deviation; a covariate with a standardized difference greater than 0.2 was considered imbalanced. | | | | | | | |  |

| Table M. Sensitivity analyses of IBD during follow-up in individuals with a lower GI biopsy result of normal mucosa, compared with their matched population references | | | | | | | | | | |
| --- | --- | --- | --- | --- | --- | --- | --- | --- | --- | --- |
| Subgroup | No. of events | | Average HR (95%CI) |  | Years since cohort entry, HR (95%CI) | | | | | |
|  | Normal mucosa | References |  |  | 6 months | 1 year | 5 years | 10 years | 20 years | 30 years |
| With a Charlson comorbidity index of zero | 4192 | 3681 | 5.92 (5.61-6.24) |  | 9.20 (8.15-10.38) | 8.18 (7.36-9.09) | 5.71 (5.33-6.12) | 4.48 (4.19-4.78) | 3.06 (2.80-3.35) | 2.45 (2.12-2.82) |
| With a number of healthcare visit of zero | 3571 | 3264 | 6.15 (5.81-6.51) |  | 9.98 (8.74-11.39) | 8.90 (7.94-9.98) | 6.12 (5.70-6.58) | 4.69 (4.36-5.04) | 3.02 (2.75-3.32) | 2.30 (1.97-2.69) |
| Without GI diseases | 2035 | 3361 | 5.37 (5.04-5.73) |  | 7.30 (6.29-8.47) | 6.87 (6.03-7.82) | 5.52 (5.11-5.95) | 4.57 (4.21-4.96) | 3.06 (2.77-3.39) | 2.34 (1.96-2.79) |
| Without endoscopy | 3777 | 3976 | 5.65 (5.36-5.97) |  | 8.09 (7.18-9.12) | 7.40 (6.67-8.22) | 5.57 (5.21-5.95) | 4.46 (4.17-4.76) | 3.03 (2.77-3.31) | 2.39 (2.07-2.76) |
| Without colectomy or proctocolectomy | 4796 | 4085 | 5.56 (5.29-5.86) |  | 8.00 (7.16-8.94) | 7.21 (6.54-7.95) | 5.31 (4.97-5.67) | 4.24 (3.99-4.51) | 3.00 (2.75-3.28) | 2.46 (2.15-2.81) |
| Individuals with index date in January 2006 or later | 633 | 874 | 3.06 (2.71-3.45) |  | 3.66 (2.92-4.59) | 3.36 (2.82-4.01) | 2.49 (2.17-2.87) | 1.85 (1.36-2.52) | - | - |
| Hazard ratio (HR) was estimated from the flexible parametric survival model. All models were conditioned on matching set (birth year, sex, county of residence, and calendar period) and further adjusted for country of birth, educational attainment, number of healthcare visits, Charlson comorbidity index, and history of GI diseases. | | | | | | | | | | |

**Reference**

1. Nguyen LH, Örtqvist AK, Cao Y, Simon TG, Roelstraete B, Song M, et al. Antibiotic use and the development of inflammatory bowel disease: a national case-control study in Sweden. The Lancet Gastroenterology & Hepatology. 2020;5(11):986-95. doi: 10.1016/s2468-1253(20)30267-3.

2. Mouratidou N, Malmborg P, Jaras J, Sigurdsson V, Sandstrom O, Fagerberg UL, et al. Identification of Childhood-Onset Inflammatory Bowel Disease in Swedish Healthcare Registers: A Validation Study. Clin Epidemiol. 2022;14:591-600. doi: 10.2147/CLEP.S358031. PMID: 35520278.

3. Everhov AH, Sachs MC, Malmborg P, Nordenvall C, Myrelid P, Khalili H, et al. Changes in inflammatory bowel disease subtype during follow-up and over time in 44,302 patients. Scand J Gastroenterol. 2019;54(1):55-63. doi: 10.1080/00365521.2018.1564361. PMID: 30700170.

4. Shrestha S, Olen O, Eriksson C, Everhov AH, Myrelid P, Visuri I, et al. The use of ICD codes to identify IBD subtypes and phenotypes of the Montreal classification in the Swedish National Patient Register. Scand J Gastroenterol. 2020;55(4):430-5. doi: 10.1080/00365521.2020.1740778. PMID: 32370571.
