# Supplementary material for: Long-term risk of inflammatory bowel disease after endoscopic biopsy with normal mucosa: A population-based, sibling-controlled cohort study in Sweden
Source: PLoS Med. 2023 Feb 23;20(2):e1004185. doi: 10.1371/journal.pmed.1004185 (PMC9949679; doi:10.1371/journal.pmed.1004185)
Supplement: S1 Text — (DOCX) [file pmed.1004185.s003.docx]

**Statistical Analysis Plan**

**Long-term risk of inflammatory bowel disease after endoscopic biopsy with normal mucosa: A population-based, sibling-controlled cohort study**

Jiangwei Sun, PhD

[Jiangwei.sun@ki.se](mailto:Jiangwei.sun@ki.se)

Jonas F. Ludvigsson, MD, PhD

[jonasludvigsson@yahoo.com](mailto:jonasludvigsson@yahoo.com)

Department of Medical Epidemiology and Biostatistics

Karolinska Institutet

Stockholm

Sweden

**Modification history**

Created by Jiangwei Sun: 2022-04-07

Updated by Jiangwei Sun: 2022-11-15 (adding non-prespecified analyses according to comments from external reviewers)

**STUDY OBJECTIVES**

To investigate the long-term risk of inflammatory bowel disease (IBD) among individuals with a gastrointestinal (GI) biopsy of normal mucosa.

**METHOD**

**Study design**

We will conduct a cohort study, based on the nationwide histopathology cohort ESPRESSO (Epidemiology Strengthened by histoPathology Reports in Sweden) ^1^. ESPRESSO contains information on all computerized GI biopsy reports from 28 pathology departments during 1965-2016 in Sweden, including date of biopsy, topography (upper GI tract: T60-T65; and lower GI tract: T66-T69 or T6X), and morphology (through a Swedish version of the Systematized Nomenclature of Medicine (SNOMED) coding)^1^. The exposed individuals will be identified as those with a first GI biopsy report of normal mucosa (SNOMED codes: M00100 and M00110) and without other aberrations earlier. Individuals with an earlier diagnosis of IBD will be excluded from the analysis.

For each exposed individual, we will randomly select up to five reference individuals from the Swedish Total Population Register^2^ by birth year, sex, county of residence, and calendar period. Reference individuals should be alive and biopsy-naïve at time of selection. We will conduct a sibling cohort to compare the risk of IBD between the exposed individuals and their unexposed full siblings (identified from the Swedish Multi-Generation Register^3^). The full siblings should have to be alive at the biopsy date of the exposed individual. Date of biopsy for the exposed individuals and date of selection for population references or unexposed full siblings will be used as the index date.

**Follow-up and ascertainment of outcome**

Study participants will be linked to the Swedish national healthcare registers, using the unique personal identity number assigned to all residents in Sweden^4^, with a virtually complete follow-up until an incident diagnosis of IBD, proctocolectomy (when UC as the outcome), emigration, death, or December 31, 2016.

We will identify newly diagnosed IBD as having one relevant International Classification of Disease (ICD) code for IBD in the National Patient Register and one biopsy record indicating IBD in the ESPRESSO (see below **Table 1** for relevant codes). IBD will be divided into three subtypes (i.e., UC, CD, or IBD-U). Due to the lower positive predictive value for IBD-U^5^, associations between normal GI mucosa and risk of IBD-U will be not explored. For normal upper GI mucosa, we will only explore the risk of CD, since UC is limited to the rectum and colon.

**Covariates**

The following covariates will be considered. We will retrieve data on country of birth from the Total Population Register^2^ (Nordic or others) and educational attainment from the Swedish Longitudinal Integrated Database for Health Insurance and Labour Market Studies^6^ (4 groups: 0-9 years, 10-12 years, ≥13 years, and “missing”). Number of non-primary healthcare visits, as a proxy for regular healthcare seeking behavior, will be defined as the number of healthcare visits between 2 years and 6 months before the index date from the National Patient Register^7^ (4 groups: 0, 1, 2-3, and ≥4). Charlson comorbidity index^8^, without considering ulcer disease and as a proxy for general health status, will be calculated according to diagnoses from the National Patient Register (3 groups: 0, 1, and ≥2). Finally, we will consider history of GI diseases before the index date according to the National Patient Register (yes/no, see below **Table 2** for ICD codes).

**STATISTICAL ANALYSES**

We will explore the risk of overall IBD, UC, and CD in individuals with a normal lower GI mucosa and risk of CD in those with a normal upper GI mucosa. Follow-up will be started from 6 months after the index date. To estimate the average and temporal pattern of hazard ratio (HR), comparing the exposed individuals to the matched population references and unexposed full siblings, flexible parametric survival model will be applied to allow the effect of normal mucosa to vary over time ^9^. Standardized cumulative incidence of IBD will be estimated using such approach^10^. Time since date of cohort entry will be used as the underlying time scale.

We will present HR as well as cumulative incidence and its difference at 6 months, 1 year, 5 years, 10 years, 20 years, and 30 years after cohort entry for each outcome. In the population-matched cohort, we will condition the analyses on the matching variables (birth year, sex, county of residence, and calendar period), and additionally adjust for other covariates (mentioned above). In the sibling cohort, we will perform similar analyses, condition on family identifier as well as adjust for birth year, sex, county of residence, calendar period, and the aforementioned covariables.

**Subgroup and sensitivity analyses**

We will stratify the analysis by sex (male or female), age at index date (<18 y, 18-39.9 y, 40-59.9 y, and ≥60 y), and calendar period at index date (1969-1989, 1990-1999, 2000-2009, and 2010-2016).

We will perform a number of sensitivity analyses to assess the robustness of our results. We will restrict the analysis to: (a). individuals with a Charlson comorbidity index of zero; (b). individuals without a healthcare visit between 2 years and 6 months before the index date; (c). individuals free of GI diseases before the index date; (d). individuals free of endoscopy (see below **Table 2** for relevant codes); and (e). individuals free of colectomy or proctocolectomy (see below **Table 2** for relevant codes). We will calculate the E-value to identity the minimum strength of the association an unmeasured confounder would need to have with both exposure and outcome to explain away the observed association^11^.

Data analyses will be performed using SAS version 9.4 (SAS Institute Inc, Cary, NC), Stata (version 16.1; StataCorp LP, College Station, TX), and R version 3.6.0. A two-sided *P* ≤ 0.05 will be considered statistically significant.

*Non-prespecified Analysis (Analysis according to comments from external reviewers):*

- To assess whether the age at index date differs between the exposed individuals and their unexposed full siblings, we added statistical comparison tests to almost all variables in Table 1.
- We added more analyses to explore the associations between a GI biopsy of normal mucosa and risk of IBD phenotypes, including CD location and UC extent. Data on CD location and UC extent were collected at the date of IBD diagnosis and classified according to the Montreal classification. CD location includes ileal (L1)/ileocolonic (L3)/unknown (LX) or colonic (L2). UC extent includes proctitis (E1)/left-sided colitis (E2), extensive colitis (E3) or extent not defined (EX).
- In the subgroup analyses by sex, age at index date, and calendar period at index date, we calculated the *P* value for interaction using the Wald test for the product terms between the exposure and subgroup variables.
- To assess the robustness of our results, we further added one sensitivity analysis by restricting the analysis to individuals with index date on 1 January 2006 or later (because in Sweden, the Prescribed Drug Register was only available since July 2005; and we wanted an exposure period of ≥6 months to minimize exposure misclassification) and without prescription of IBD medication before index date.
- Standardized difference was used to examine the balance of a covariate between the exposed and unexposed groups and imbalance was defined as a standardized difference value greater than 0.2.

| Table 1. International Classification of Disease (ICD) codes and SNOMED codes defining inflammatory bowel diseases (IBD) ^a^. | | | | | |
| --- | --- | --- | --- | --- | --- |
| IBD subtypes | ICD-7 (1964-1968) | ICD-8 (1969-1986) | ICD-9 (1987-1996) | ICD-10 (1997-) | SNOMED codes ^b^ |
| Ulcerative colitis (UC) | 572,20; 572,21; 578,03 | 563,1; 563,10; 569,02; 569,04 | 556 | K51 | D6255 or M41, M42, M43, M44, M463, or M47 |
| Crohn's disease (CD) | 572,00; 572,09 | 563,00 | 555 | K50 | D6216 or M41, M42, M43, M44, M463, or M47 |
| IBD unclassified (IBD-U) | UC + CD | UC + CD or 563; 563,0; 563,9; 563,98; 563,99 | UC + CD | UC + CD or K52.3 | D6214 or M41, M42, M43, M44, M463, or M47 |
| ^a^ Subtypes of IBD were defined according to the first two diagnostic codes only, therefore no information after start of follow-up contributed to such definition; and the IBD subtype was only determined by the ICD code if one individual had one ICD code for IBD and one M code. | | | | | |
| ^b^ SNOMED codes starting with M (inflammation suggestive of IBD, but not a specified subtype) were required to be accompanied by a topographic code of T67 or T68 (colon); for example, M41 refers to all codes starting with M41; D codes are diagnostic codes but listed under morphology in pathology registers; D6255 for example is the diagnostic code for UC. | | | | | |

| Table 2. Definitions of endoscopy, colectomy, and proctocolectomy | | |
| --- | --- | --- |
| Procedures | Classification of procedures | Definition code |
| Endoscopy | Esophagogastroduodenoscopy | 2861, 2880, 2881, 4480, 4483, 4486, 4487, 4488, 4489, 4490, 9021, 4686, 4687, 9003, 9004, 9021, UJC, UJD, UJF02, UJF05 |
|  | Colonoscopy or sigmoidoscopy | 9011, 9012, 9023, 4685, 4688, 4689, 4674, 4684, UJF32, UJF35, UJF42, UJF45 |
| Colectomy | Sixth revision | 4650, 4651 |
|  | Seventh revision | JFH10, JFH11, JFH96, JFH00, JFH01, JFC40, JFC41, JFG29, JFG26 |
| Proctocolectomy | Sixth revision | 4652, 4653, 4654 |
|  | Seventh revision | JFH30, JFH33, JGB50, JGB60, JFH40, JFH20 |
| Gastrointestinal disease | ICD-7 (1964-1968) | 530-587 |
|  | ICD-8 (1969-1986) | 520-579 |
|  | ICD-9 (1987-1996) | 520-579 |
|  | ICD-10 (1997-) | K00-K99 |

**Reference:**

1. Ludvigsson JF, Lashkariani M. Cohort profile: ESPRESSO (Epidemiology Strengthened by histoPathology Reports in Sweden). *Clin Epidemiol.* 2019;11:101-114.

2. Ludvigsson JF, Almqvist C, Bonamy AK, et al. Registers of the Swedish total population and their use in medical research. *Eur J Epidemiol.* 2016;31(2):125-136.

3. Ekbom A. The Swedish multi-generation register. *Methods in biobanking*: Springer; 2011:215-220.

4. Ludvigsson JF, Otterblad-Olausson P, Pettersson BU, Ekbom A. The Swedish personal identity number: possibilities and pitfalls in healthcare and medical research. *Eur J Epidemiol.* 2009;24(11):659-667.

5. Shrestha S, Olen O, Eriksson C, et al. The use of ICD codes to identify IBD subtypes and phenotypes of the Montreal classification in the Swedish National Patient Register. *Scand J Gastroenterol.* 2020;55(4):430-435.

6. Ludvigsson JF, Svedberg P, Olen O, Bruze G, Neovius M. The longitudinal integrated database for health insurance and labour market studies (LISA) and its use in medical research. *Eur J Epidemiol.* 2019;34(4):423-437.

7. Ludvigsson JF, Andersson E, Ekbom A, et al. External review and validation of the Swedish national inpatient register. *BMC Public Health.* 2011;11:450.

8. Ludvigsson JF, Appelros P, Askling J, et al. Adaptation of the Charlson Comorbidity Index for Register-Based Research in Sweden. *Clin Epidemiol.* 2021;13:21-41.

9. Lambert PC, Royston P. Further development of flexible parametric models for survival analysis. *Stata J.* 2009;9(2):265-290.

10. Hinchliffe SR, Lambert PC. Flexible parametric modelling of cause-specific hazards to estimate cumulative incidence functions. *BMC Med Res Methodol.* 2013;13:13.

11. VanderWeele TJ, Ding P. Sensitivity Analysis in Observational Research: Introducing the E-Value. *Annals of internal medicine.* 2017;167(4):268-274.
