# Supplementary material for: Long-term risk of inflammatory bowel disease after endoscopic biopsy with normal mucosa: A population-based, sibling-controlled cohort study in Sweden
Source: PLoS Med. 2023 Feb 23;20(2):e1004185. doi: 10.1371/journal.pmed.1004185 (PMC9949679; doi:10.1371/journal.pmed.1004185)
Supplement: Attachment — Submitted filename: R1_letter to editor and reviewers_final.docx [file pmed.1004185.s004.docx]

**Requests from the editors:**

Please structure your abstract using the PLOS Medicine headings (Background, Methods and Findings, Conclusions).

**Reply:** The abstract has now been modified accordingly.

Abstract Background: Provide the context of why the study is important. The final sentence should clearly state the study question.

**Reply:** We have now modified the Background accordingly.

On page 4 (page number in the version with track changes):

*“Although evidence suggests a persistently decreased risk of colorectal cancer for up to 10 years among individuals with a negative endoscopic biopsy result (i.e., normal mucosa), concerns have been raised about other long-term health outcomes among these individuals. In this study, we aimed to explore long-term risk of inflammatory bowel disease (IBD) after an endoscopic biopsy with normal mucosa.”*

In the last sentence of the Abstract Methods and Findings section, please describe the main limitation(s) of the study's methodology.

**Reply:** Thank you for the suggestion. We have now stated the main limitations of the study in the Methods and Findings as follows:

On page 5:

*“Study limitations include lack of data on indications for biopsy and potential residual confounding from unmeasured risk or protective factors for IBD.”*

At this stage, we ask that you include a short, non-technical Author Summary of your research to make findings accessible to a wide audience that includes both scientists and non-scientists. The Author Summary should immediately follow the Abstract in your revised manuscript. This text is subject to editorial change and should be distinct from the scientific abstract.

**Reply:** We appreciate your suggestion. The “Author summary” section has now been added after the abstract. Please also see below:

On page 6:

*"****Author summary***

***Why was this study done?***

- *As the most frequent gastrointestinal (GI) histologic finding on endoscopy, a negative endoscopic result (i.e., normal mucosa) has been associated with a persistently decreased risk of colorectal cancer for up to 10 years. However, concerns have been raised about other long-term health outcomes among these individuals.*
- *Until now, the long-term risk of inflammatory bowel disease (IBD) after an endoscopic biopsy with normal mucosa remains unclear.*

***What did the researchers find?***

- *Compared with population references and unexposed full siblings, individuals with a lower GI biopsy of normal mucosa had a persistently higher risk of overall IBD, ulcerative colitis (UC), and Crohn’s disease (CD).*
- *Individuals with an upper GI biopsy of normal mucosa were also at an increased risk of CD.*
- *The elevated risk of IBD persisted at least 30 years after a biopsy with normal mucosa.*

***What do these findings mean?***

- *Our findings signal a substantial symptomatic period before IBD diagnosis.*
- *Clinicians should be aware of the long-term increased risk of IBD in those with symptoms requiring GI investigation but with a finding of histologically normal mucosa."*

Please ensure that the study is reported according to the STROBE guideline, and include the completed STROBE checklist as Supporting Information. Please add the following statement, or similar, to the Methods: "This study is reported as per the Strengthening the Reporting of Observational Studies in Epidemiology (STROBE) guideline (S1 Checklist)."When completing the checklist, please use section and paragraph numbers, rather than page numbers.

**Reply:** We appreciate your suggestion and have now added the STROBE checklist accordingly, referred to it in the Methods, and attached it as a supplementary file (S1 Checklist).

On page 13:

*“This study is reported as per the Strengthening the Reporting of Observational Studies in Epidemiology (STROBE) guideline (S1 Checklist)”*

Did your study have a prospective protocol or analysis plan? Please state this (either way) early in the Methods section.

**Reply:** Thank you for the suggestion. We have now stated about the pre-specified analysis plan in Method, added it as a supplementary file (S1 Text), and highlighted analyses that were not pre-specified.

On page 9:

*“The pre-specified analysis plan is presented in S1 Text.”*

For the adjusted analyses in Tables 2 and 3, please also provide the unadjusted analyses.

**Reply:** Because of the individually matched design of the cohort study, all results are inherently adjusted for matching factors (birth year, sex, county of residence, and calendar period). We can therefore not present completely unadjusted results. However, we are certainly willing to reconsider our position if the editor does not agree with us.

Please include definitions of the abbreviations used in Tables 2 and 3 in the legend.

**Reply:** We have now defined the abbreviations in the footnotes of Table 2 (on page 18) and Table 3 (on page 22) accordingly.

In Figure 1 panels A through D, please ensure that the Y axis is identical for all figures to facilitate comparison.

**Reply:** We have now modified the panels A-D accordingly.

**Comments from the reviewers:**

**Reviewer #1:**

The manuscript entitled "Long-term risk of inflammatory bowel disease after endoscopic biopsy with normal mucosa: A population-based, sibling-controlled cohort study" present interesting results describing the implication of normal mucosal biopsies and risk of IBD in a large Swedish population. Higher risk of IBD was observed in the population with a lower gastrointestinal (GI) biopsy of normal mucosa compared to a matched control population or compared to unexposed full siblings. Similar observation was obtained in individuals with an upper GI biopsy of normal mucosa. The authors suggest the existence of a substantial preclinical period of IBD and incomplete diagnostic examinations in patients with early IBD.

**Reply:** We appreciate your positive assessment.

**Methods**

-The authors mentioned that "For normal upper GI mucosa, only the risk of CD was assessed, since UC is limited to the rectum and colon." While true, the study missed the opportunity to demonstrate the negative predictive value of UC diagnosis of the International Classification of Disease coding system, which would overall reinforce the strength of this coding used in the study.

**Reply:** Thank you for the comment. However, we did not have such data to calculate the negative predictive value of UC. This would require us to examine patient charts in a large number of individuals with normal mucosa (i.e. negative for IBD), but we did not have access to such patient charts. According to our previous study, the approach that was used to identify IBD in the current study has a positive predictive value of 95% (95%CI: 89%-99%) [**Ref 1-2**].

**Ref 1:** Nguyen LH et al. The Lancet Gastroenterology & Hepatology. 2020;5(11):986-95.

**Ref 2:** Mouratidou N et al. Clin Epidemiol, 2022, 14: 591-600.

- It is unclear how the matching of the population of references was selected. A large amount of covariate are presented, and it's not clear if those covariate had equal weight in the matching process. Can you clarify?

**Reply:** Thank you for your comment. In the present study, for each exposed individual with a GI biopsy of normal mucosa, we randomly selected up to five reference individuals from the general population with individual matching by birth year, sex, county of residence, and calendar period. Other covariates were not considered in the matching procedure. We have now clarified the matching procedure in the Methods.

On page 9:

*"In a population-matched cohort, for each exposed individual, we randomly selected up to five reference individuals from the Swedish Total Population Register [27], who were individually matched to the exposed patient by birth year, sex, county of residence, and calendar period. Reference individuals should be alive and biopsy-naïve at date of selection."*

-It seems that the author had access to the general population and not only the matched reference population. Generally speaking, a regression model applied to the entire cohort is often a more powerful tool in detecting a given effect as compared to a matched study (PMC4756459). Can the author justify the choice to select a match-control design?

**Reply:** Thanks for drawing our attention to the paper by Brazauskas et al. and we read it with interest. However, we do not have access to all data of the general population (and will not be allowed such access by the Ethics Review Authority due to the large number of individuals already involved in ESPRESSO) [**Ref 1**]. We conducted this study based on the nationwide histopathology cohort ESPRESSO. When constructing ESPRESSO, the person that had a biopsy was matched up to five references that were randomly selected from the general population for birth year, sex, county of residence, and calendar year. Therefore we applied a matched cohort study design in our study.

**Ref 1:** Ludvigsson J F et al. Clin Epidemiol, 2019, 11: 101-114.

-It seems that a "flexible parametric survival model" was used, then the authors mentioned that "In the population-matched cohort, we conditioned the analyses on the matching variables (birth year, sex, county of residence, and calendar period). It's not clear what package was used or if the analysis used Cox regression or if conditional logistic regression was used to account for the matching process. Can you clarify?

**Reply:** Thank you for your comment. We used the flexible parametric survival model with

the stpm2 command in Stata and have now clarified this in the Statistical analyses. Cox regression and conditional logistic regression were not used in our study.

On page 11:

*" To estimate the average and temporal pattern of hazard ratio (HR), with 95% confidence interval (CI), comparing the exposed individuals to the matched population references and unexposed full siblings, flexible parametric survival model using the stpm2 command in Stata was applied to allow the effect of normal mucosa to vary over time (a time-varying effect)[39]. "*

-For the Sibling comparison, one of the adjusted variables was the analysis on the county of residence. Since adult's sibling may not anymore live in the same household, what was the timepoint selected for this variable?

**Reply:** County of residence was collected before index date (i.e., date of biopsy for the exposed individuals and date of selection for unexposed full siblings). We have now clarified this in the Statistical analyses.

On page 12:

*"In the sibling cohort, we performed similar analyses and conditioned on family identifier as well as adjusted for birth year, sex, county of residence (collected before index date), calendar period, and the aforementioned covariables."*

- It seems that the authors have access to pedigree data. Was family history of IBD available in this cohort? Since healthy subject with multiple family members affected with IBD are at higher risk to develop IBD, it remains possible that the increased risk of IBD for subject with normal mucosa is confounded by being part of a multiplex.

**Reply:** Thank you for your comment. Those factors mentioned by the reviewer are more likely to cluster within families, we therefore conducted a sibling comparison to relieve such concerns. We observed similar results from the sibling comparison, arguing against that such concerns are a big issue in our study. We have mentioned it in the Methods and Discussion.

Methods, on page 9:

*"To assess the influence of residual confounding from genetics and early environmental factors shared within families[28], we also conducted a sibling cohort to compare the risk of IBD between the exposed individuals and their unexposed full siblings."*

Discussion, on page 27:

*"Moreover, sibling comparisons that yielded similar results helped allay concerns about potential residual confounding from genetics and early environmental factors shared within families."*

**Results**

-Table 1 would benefit from a statistical comparison between the unexposed full siblings and sibling with normal mucosa. Was there an age of diagnosis difference between the unexposed full siblings and sibling with normal mucosa?

**Reply:** Thank you for your comment. We have now added the statistical comparison test in Table 1 (on pages 15-16). And we found the age between exposed individuals and unexposed full siblings was statistically significantly different.

-The comparison and results with the unexposed siblings are interesting. Did the authors compared if the age difference between sibling contribute to an increased risk of IBD? Recent publication from Spencer et al. 2020 suggests a "clustering" of IBD onset in sibling born close to each other. This study could confirm if this is the case here as well, especially given the large sample size available here.

**Reply:** Thanks for drawing our attention to the paper by Spencer et al. and we read it with interest. We have not looked at whether age difference between siblings contribute to risk of IBD, because we feel that is not the purpose of our study.

However, inspired by this paper, we explored the risk of IBD by comparing the exposed individuals with 1). Siblings with age difference < 4 years and 2). Siblings with age difference ≥ 4years. We applied 4 years here, since in Spencer’s paper, the median age difference between affected siblings was 4 years.

From below Table 1, the risks of IBD were similar when compared to siblings with age difference <4 years and when compared to siblings with age difference ≥ 4 years (such as 3.27 vs. 3.45), and there were no statistically significant differences. Again, since this was not the purpose of our study, we chose not to present the new result in our manuscript.

| Table 1. IBD during follow-up in individuals with a GI biopsy result of normal mucosa, compared with IBD-free full siblings | |
| --- | --- |
|  | HR (95%CI) |
| Normal lower GI mucosa and IBD |  |
| Compared to siblings with age difference < 4 years | 3.27 (2.98-3.58) |
| Compared to siblings with age difference ≥ 4 years | 3.45 (3.16-3.77) |
| Normal upper GI mucosa and CD |  |
| Compared to siblings with age difference < 4 years | 1.77 (1.61-1.95) |
| Compared to siblings with age difference ≥ 4 years | 1.81 (1.65-1.98) |
| Hazard ratio (HR) was estimated from the flexible parametric survival model. All models were conditioned on family identifier and adjusted for birth year, sex, county of residence, calendar period, country of birth, educational attainment, number of healthcare visits, Charlson comorbidity index, and history of GI diseases. | |

-Was disease phenotype data available in the cohort? If yes it would be interesting to compare the different disease behavior and location.

**Reply:** We appreciate your suggestion. We have now added the analyses to explore the associations between a lower GI biopsy of normal mucosa and risk of IBD phenotypes (including CD location and UC extent) [**Ref 1**], and added the ICD codes assigned for phenotypes of IBD in **eTable 3**, and added the new result in **eTable 11** in the supplementary materials. We have updated the Methods and Results of the main text accordingly.

Methods, on page 12:

*"For normal lower GI mucosa, the risk of disease phenotypes, including CD location and UC extent, were also explored. Data on CD location and UC extent were collected at the date of IBD diagnosis and classified according to the Montreal classification [34]. CD location includes ileal (L1)/ileocolonic (L3)/unknown (LX) or colonic (L2). UC extent includes proctitis (E1)/left-sided colitis (E2), extensive colitis (E3) or extent not defined (EX) (see* ***eTable 3*** *for ICD codes). "*

Results, on pages 20-21:

*"Positive associations were also noted between normal lower GI mucosa and risk of different IBD phenotypes, such as colonic CD (average HR= 4.09 (3.24-5.17)) and UC with extensive colitis (average HR= 4.94 (4.09-5.96)) (****eTable*** ***11****)."*

**Ref 1:** Shrestha S et al. Scand J Gastroenterol, 2020, 55(4): 430-435.

-Was the time to disease onset similar across the unaffected sibling and the matched control population? I suspect full sibling to have more awareness of IBD and thus they might be more likely to have an earlier diagnosis than the general population.

**Reply:** Thank you for your comment. Compared with reference individuals of the individual with a GI biopsy of normal mucosa, the unexposed full siblings tend to have shorter years to be diagnosed with IBD since the index date (see below Table 2). However, direct comparison between reference individuals and unexposed full siblings was not the purpose of our study, we chose not to present the new result in our manuscript.

| Table 2. Comparison between reference individuals and unexposed full siblings | | | | |
| --- | --- | --- | --- | --- |
|  |  | Reference individuals | Unexposed full siblings | P |
| Lower GI biopsy and IBD | | | | |
| Age at index date, years | Mean ± SD | 37.1 ± 18.7 | 35.4 ± 16.2 | <0.001 |
|  | Median (IQR) | 35.6 (22.5-51.2) | 34.4 (23.0-46.9) |  |
| Years from index date to disease onset | Median (IQR) | 8.5 (3.9-14.9) | 7.6 (3.3-14.1) | <0.001 |
| Lower GI biopsy and UC | | | | |
| Age at index date, years | Mean ± SD | 36.4 ± 18.4 | 35.3 ± 15.3 | 0.089 |
|  | Median (IQR) | 34.5 (22.4-49.8) | 34.2 (24.5-45.7) |  |
| Years from index date to disease onset | Median (IQR) | 8.5 (3.6-14.9) | 7.6 (3.1-13.7) | 0.005 |
| Lower GI biopsy and CD | | | | |
| Age at index date, years | Mean ± SD | 36.3 ± 18.5 | 34.0 ± 16.5 | 0.011 |
|  | Median (IQR) | 35.0 (21.8-50.3) | 32.8 (21.4-47.0) |  |
| Years from index date to disease onset | Median (IQR) | 8.2 (3.9-14.4) | 7.1 (3.1-13.6) | 0.079 |
| Upper GI biopsy and CD | | | | |
| Age at index date, years | Mean ± SD | 38.8 ± 18.2 | 37.0 ± 16.3 | 0.036 |
|  | Median (IQR) | 37.8 (23.8-52.5) | 37.2 (23.8-49.9) |  |
| Years from index date to disease onset | Median (IQR) | 7.1 (3.3-12.5) | 7.2 (3.3-12.7) | 0.948 |
| CD: Crohn's disease; GI: gastrointestinal; IBD: inflammatory bowel disease; IQR, interquartile range; SD, standard deviation; UC: ulcerative colitis. | | | | |
| Index date was defined as the date of selection for reference individuals and unexposed full siblings. | | | | |

**Discussion**

-When describing unmeasured risk factors, the authors mentioned that "Such concern however might be relieved partly by the similar results from the sibling comparison, as these factors are more likely to cluster within families." This not completely true for many of the described risk factor including oral contraceptive use (sibling might be of a different a different sex), or current lifestyle factors (the sibling may not be currently living in the same household).

**Reply:** We have removed "oral contraceptive use" from the sentence and revised the sentence to make it more precise.

On pages 27-28:

*"Third, due to the register-based nature of the study, we did not have complete information on all protective or risk factors for IBD, including lifestyle factors (e.g., smoking, body-mass index, and physical activity)[5,45], medical conditions (e.g., antibiotic exposure and vitamin D deficiency)[5,32], and genetic factors (e.g., ethnicity[1,46] and risk loci (e.g., NOD2 in European populations)[47,48]), leaving residual confounding as a concern. However, such concern might be relieved partly by the similar results from the sibling comparison. On the other hand, since genetic and early environmental factors as well as IBD are more likely to cluster within families [49], some siblings may also have undiagnosed IBD, making effect estimates in sibling comparison too conservative."*

**Reviewer #2:**

The paper by Sun et al presents a retrospective cohort study of people who have had a GI biopsy, showing normal mucosa, matched to unexposed individuals both from the general population and from their siblings. The paper reports increased rates of IBD in the exposed group, with risk increased for as much as 30 years after the initial biopsy. This review considers the use of statistics in the paper.

The general approach of using flexible parametric survival models is appropriate, since it allows modelling of time-varying associations between exposures and outcomes, and extraction of hazard functions and ratios, and cumulative incidence, for specific subgroups. This works well for this paper.

**Reply:** We appreciate the positive assessment of the peer reviewer.

One comment: is it strictly necessary to include a six-month blanking period after the index date, if the model allows for time-varying hazards? Maybe it is - it's just a thought.

**Reply:** We appreciate your comment. In the main analysis, we chose to start the follow up from the six months after the index date mainly aiming at decreasing potential detection bias (e.g., work up for GI biopsy increases the chance of diagnosing the other), surveillance bias (e.g., regular check-ups after a GI biopsy increase chance of early detection of IBD), and reverse causation. We have now clarified this in the Methods.

Methods, on page 11:

*"Follow-up (i.e., date of cohort entry) was started 6 months after the index date to decrease potential detection bias (e.g., work up for GI biopsy increases the chance of diagnosing the other), surveillance bias (e.g., regular check-ups after a GI biopsy increase chance of early detection of IBD), and reverse causation."*

The following comments are quite minor.

The title mentions the use of sibling controls, but not the general population controls.

**Reply:** Thank you for the comment. The term "population-based, sibling-controlled" in the current title means "population-based cohort study and sibling-controlled cohort study". If the reviewer or editor suggests a different title, we would be happy to discuss another title.

The methods section of the abstract talks only of lower GI biopsy, though the study does look at upper GI biopsy as well. This does not become clear until later in the paper.

**Reply:** We have now added the information on upper GI biopsy accordingly.

On page 4:

*"In the present nationwide cohort study, we identified all individuals in Sweden with a lower or upper gastrointestinal (GI) biopsy of normal mucosa during 1965-2016 (exposed, n=200,495 and 257,192 for lower and upper GI biopsy, respectively), their individually matched population references (n=989,484 and 1,268,897), and unexposed full siblings (n=221,179 and 274,529)."*

On page 5:

*"For individuals with an upper GI biopsy of normal mucosa, the average HR of CD was 2.93 (2.68-3.21) and 2.39 (2.10-2.73), compared with population references and unexposed full siblings, respectively."*

The conclusion of the abstract talks of elevated risk for at least 30 years. This is conclusion is reasonable, give the results reported in the paper as a whole, but not based on the results reported in the abstract, which shows only average HRs.

**Reply:** Thank you for the comment. We have now added such information in the Abstract.

On page 5:

*"The increased risk of IBD persisted at least 30 years after cohort entry."*

In the abstract, and possibly in the paper as a whole, not much is made of the difference in results between using general population controls and sibling controls. I think it is important to note how much of the general population association can be explained by shared genetic and/or environmental factors, or whatever it is that is causing this difference.

**Reply:** We appreciate your comment and have now commented this in the Discussion.

On page 28:

*"On the other hand, since genetic and early environmental factors as well as IBD are more likely to cluster within families[49], some siblings may also have undiagnosed IBD, making effect estimates in sibling comparison too conservative."*

I am sure this is the case, but I assume the Charlson scores were derived only from data available prior to the index date?

**Reply:** Charlson comorbidity score was indeed derived before the index date. We have now clarified this in the Methods.

On page 11:

*"Charlson comorbidity index [37] before the index date, without considering ulcer disease and as a proxy for general health status, was calculated according to diagnoses from the National Patient Register (3 groups: 0, 1, and ≥2)."*

A number of subgroup analyses are performed, by stratifying the analysis. This is OK, but if possible, I prefer to see subgroup analyses in terms of a single model with an interaction between the subgroup and the exposure variables. Otherwise, the effects of other covariates can differ between the separate models, but these differences are not assessed - only the variation in association between exposure and outcome are reported. However, I accept there are different views on this topic, so it is not critical.

**Reply:** We agree with the reviewer and have now added an interaction test and the relevant *P* value for the interaction test in the Methods and Results, respectively.

Methods, on page 12:

“The *P* value for interaction was calculated using the Wald test for the product terms between the exposure and subgroup variables.”

Results, on page 20:

*"In subgroup analyses by sex, age, and calendar period, the HR for overall IBD was higher in males (average HR=6.24 (5.77-6.76)) than females (5.11(4.78-5.46)), and in individuals aged 18-39.9 years (6.38 (5.91-6.88)) than other age groups in the population-matched cohort (both P _for interaction_ < 0.001,* ***eTable 9*** *for HR,* ***eFigure 1*** *for cumulative incidence). "*

*"In analyses of overall IBD in relation to calendar period of biopsy, the HR and cumulative incidence were lower during the latter two calendar periods (P _for interaction_ < 0.001), for example within 5 years after cohort entry (****Figure 3,*** ***eTable 9****,* ***and*** ***eFigure 1****), compared with earlier calendar periods. "*

Would it be appropriate to cite the specific package(s) used to fit these models (if done in R)?

**Reply:** We used the flexible parametric survival model with the stpm2 command in Stata and have now clarified it in the Statistical analyses.

On page 11:

*" To estimate the average and temporal pattern of hazard ratio (HR), with 95% confidence interval (CI), comparing the exposed individuals to the matched population references and unexposed full siblings, flexible parametric survival model using the stpm2 command in Stata was applied to allow the effect of normal mucosa to vary over time (a time-varying effect)[39]. "*

Reporting the number of additional cases per exposed individual is an interesting way to look at the data. Viewed in this way, how many of these could simply be thought of as false negatives amongst the original biopsies? Does this align with the reducing HRs over time, assuming that the false negative rate has improved?

**Reply**: We appreciate your interesting comment. However, since we did not have data to show how many individuals with a GI biopsy of normal mucosa actually had *undiagnosed and unrecorded* IBD before being diagnosed with IBD, we are afraid that we cannot test this hypothesis raised by the reviewer.

Personally, I do not thing the E-value adds very much. It says how strong an association would need to be with a single covariate to explain the observed association, but in reality there may be many unknown confounders. I think most readers can interpret the magnitude of the observed association without this.

**Reply**: Thank you for your comment on E-value. We agree with the reviewer that there may exist many unknown confounders, and unmeasured confounding from one or even multiple unknown confounders is considered as one of the central challenges in observational studies. This is the main reason why we reported E-value in our study, since E-value assesses robustness to unmeasured confounding. Reporting it would help readers see how large a hypothetical unmeasured confounding would be needed to explain away the observed association in our study. However, we are certainly willing to reconsider our position if the reviewer or editor does not agree with us.

The language used throughout the paper implies that receiving a normal biopsy is a risk factor for future IBD. Whilst I am sure this is not the intention, this seems contradictory. Regardless of the causal process involved (and regardless of the E-value reported) there must be unmeasured factors that explain the observed association. For example, are the authors suggesting that if the people in the control groups of this analysis were given a biopsy (and received a normal result) at the same time as their matched exposed individual, then their risk of IBD would have been elevated? This could only be true if the act of doing a biopsy is a causal factor in the development of IBD.

**Reply**: We appreciate your thoughtful comment, and we have now edited the manuscript thoroughly so as not to convey the message that receiving a normal biopsy is a risk factor for future IBD.

On the selection of unexposed individuals, the methods section states that they were biopsy-naïve at the index date. Does this mean that a matched control could become an exposed individual at a later date? I think that would be important, to avoid an immortal time bias (if that is the correct term), and could be made more explicit if that is the case.

**Reply**: Thank you for your comment. Yes, some of matched reference individual could become an exposed individual if they received a GI biopsy of normal mucosa during follow-up. We have now clarified this in the Methods.

On page 10:

*"Some of reference individuals or biopsy-naïve full siblings were also censored when receiving a GI biopsy of normal mucosa during follow-up."*

**Reviewer #3:**

Thank you for inviting me to read this interesting paper on rates of IBD diagnosis following normal mucosal biopsy. It is in fact a study I would have liked to do myself if I had the time, so I was very interested to read your findings and I hope it is published after revision.

The authors used a histopathology database to identify individuals with normal colorectal mucosal biopsies and then followed them longitudinally to determine the subsequent incidence of IBD. This was 2.4% and is in itself an interesting finding. They then compared the incidence rate of IBD to a matched referent group and also a sibling cohort, and found relatively speaking that those with previous normal biopsies still had an increased risk of subsequent IBD. Please see my comments listed below. I hope they will help improve the manuscript.

**Reply:** We appreciate the positive assessment of the peer reviewer.

1. "Blackwell et al demonstrated that patients with IBD often had GI symptoms five years before biopsy" - I believe this study was from a large primary care dataset which did not have access to biopsy results and this should actually read "diagnosis" instead of "biopsy".

**Reply:** Thanks for drawing our attention to this issue and we have now edited our text.

On page 7:

*"Blackwell et al demonstrated that patients with IBD often had GI symptoms five years before diagnosis[8]."*

2. "an endoscopic finding of normal mucosa might still entail increased risk of adverse health outcomes, including neurological and psychiatric disorders, chronic obstructive pulmonary disease, non-GI cancers, and mortality." - "Entail increased risk" suggests having a biopsy causes that risk, rather than just being a non-causative association. This wording is too strong and needs to be changed. I assume this is only because of a bias in healthcare utilization among patients with more comorbidities (sick patients see doctor for one problem and mention an unrelated problem which results in more healthcare utilization including colonoscopy).

**Reply:** Thanks for your comment and we have now edited this sentence.

On pages 7-8:

*"Although evidence suggests a persistently decreased risk of colorectal cancer (CRC) for up to 10 years among individuals with a negative endoscopic result[17-19], an endoscopic finding of normal mucosa has been linked to excess risks of adverse health outcomes, including neurological and psychiatric disorders[20-22], chronic obstructive pulmonary disease[23], non-GI cancers[24], and mortality[25]."*

This is also a potential weakness in this study - are these patients more likely be diagnosed with IBD because of a selection bias? They have already demonstrated they are more likely than average population to see a doctor? Are they by definition they are a population willing to undergo endoscopy, which in the referent group is not necessarily the case? Therefore are the results in part a reflection of increased utilization of healthcare?

**Reply:** We appreciate your comment. However, we thought such concerns are unlikely to fully explain our results, based on several assumptions:

- In the main analysis, we started the follow-up from six months after the index date to decrease the risk of detection bias (e.g., work up for GI biopsy increases the chance of diagnosing the other), surveillance bias (e.g., regular check-ups after a GI biopsy increase chance of early detection of IBD), and reverse causation.
- We adjusted for Charlson comorbidity index (as a proxy for general health status), number of healthcare visit (as a proxy for regular healthcare seeking behavior), and history of GI diseases in all analyses and we observed similar results after excluding individuals with comorbidity or GI diseases before GI biopsy.
- A constantly positive association between normal mucosa and IBD was noted even at 30 years of follow-up after date of cohort entry.

We have now clarified them in the Methods and Discussion sections.

Methods, on page 11:

*"Follow-up (i.e., date of cohort entry) was started 6 months after the index date to decrease potential detection bias (e.g., work up for GI biopsy increases the chance of diagnosing the other), surveillance bias (e.g., regular check-ups after a GI biopsy increase chance of early detection of IBD), and reverse causation."*

Discussion, on page 27:

*"First, lack of data on indications for biopsy is a concern. Normal mucosa might be a surrogate of a diverse range of GI symptoms or diseases before biopsy, which may be linked to IBD. Indeed, we found that individuals with normal mucosa were more likely to have a diagnosis of functional GI diseases (e.g., irritable bowel syndrome or functional dyspepsia) five years before biopsy in a previous study[22]. This is however unlikely to explain our results, as we noted a constantly positive association between normal mucosa and IBD during the entire 30 years of follow-up. Moreover, such concern might be partly alleviated by adjusting for Charlson comorbidity index, number of healthcare visit, and history of GI diseases in the analyses and by the similar results obtained after excluding individuals with comorbidity or GI diseases before GI biopsy."*

3. "The exposed individuals were identified as those with a first GI biopsy report of normal mucosa". Is there any way of ensuring this was the patient's first ever GI biopsy, not just their first recorded biopsy in Espresso? My understanding is that Espresso is not a comprehensive database for Sweden as it covers only 28 departments. Google tells me there are 100 hospitals in Sweden. It is therefore very possible some of the small number of patients who later develop IBD were previously diagnosed elsewhere, were then treated, moved to a different hospital, and had a surveillance colonoscopy after with normal biopsies. This means it is possible these normal biopsies do not reflect pre-inflammatory IBD. If this limitation cannot be addressed it should be listed in the limitations.

**Reply:** We appreciate your comment. Although there are more than 100 hospitals in Sweden, there are only 28 pathology departments in Sweden and we collected all GI histopathology report data from those 28 departments in ESPRESSO [**Ref 1**]. Therefore ESPRESSO is a nationwide GI histopathology cohort. However, we acknowledge that a small number of biopsies may have taken place prior to computerization of the pathology departments. We have now commented this in the Discussion.

On page 27:

*"However, we cannot rule out a small number of earlier biopsies, prior to computerization of Swedish pathology departments."*

**Ref 1:** Ludvigsson J F et al. Clin Epidemiol, 2019, 11: 101-114.

4. Do you have access to the indications for the biopsies? If so, a sensitivity analysis excluding any with "surveillance" or a previous diagnosis of IBD would be useful.

**Reply:** We did not have indication data for the biopsies and have listed this as a limitation in the Discussion (please see our reply to your second comment).

We also have conducted a series of sensitivity analyses by excluding potential causes for surveillance bias from the analyses (clarified in the Methods), and observed robust results (clarified in the Results).

Methods, on page 13:

*"A number of sensitivity analyses were performed to assess the robustness of our results to potential influence from comorbidity and healthcare utilization.* *We restricted these analyses to: (a). individuals with a Charlson comorbidity index of zero; (b). individuals without a healthcare visit between 2 years and 6 months before the index date; (c). individuals free of GI diseases before the index date; (d). individuals free of previous endoscopy (see* ***eTable 2*** *for relevant codes); and (e). individuals free of previous colectomy or proctocolectomy (see* ***eTable 2*** *for relevant codes)."*

Results, on page 23:

*"The sensitivity analyses showed consistently higher risk of overall IBD among individuals with a normal lower GI mucosa, compared with the population references (****eTable 13****). The corresponding average HR was 5.92 (5.61-6.24) after restricting the analysis to individuals with a Charlson comorbidity score of zero, 6.15 (5.81-6.51) in individuals without an earlier healthcare visit, 5.37 (5.04-5.73) in those without earlier record of GI diseases, 5.65 (5.36-5.97) in those without earlier record of endoscopy, 5.56 (5.29-5.86) in those without previous colectomy or proctocolectomy, and 3.06 (2.71-3.45) in those with index date in January 2006 or later and without prescription of IBD medications before index date. Of note, although the HRs decreased in magnitude over time in all sensitivity analyses, all HRs remained statistically significant during the entire follow-up."*

Individuals with a previous diagnosis of IBD were excluded from all analyses in our study. We have clarified this in the Methods.

On page 9:

*"Individuals with an earlier diagnosis of IBD were excluded from all analyses."*

5. Do you have access to prescriptions for these patients? If so, a sensitivity analysis excluding any patients with use of any IBD drugs (mesalazine, thiopurines, systemic corticosteroids etc) before first colonoscopy would be really valuable.

**Reply:** We have now added a sensitivity analysis where we excluded individuals using any IBD medications before a GI biopsy of normal mucosa, and added the Anatomical Therapeutic Chemical (ATC) codes representing IBD medication in **eTable 4** and the new result in **eTable 13** in the supplementary materials. We have updated the Methods and Results of the main text accordingly.

Methods, on page 13:

*"* *Given that the Prescribed Drug Register was available since July 2005, we restricted the analysis to individuals with an index date in January 2006 or later and without prior prescription of IBD medication (see* ***eTable 4*** *for relevant codes)."*

Results, on page 23:

*"The sensitivity analyses showed consistently higher risk of overall IBD among individuals with a normal lower GI mucosa, compared with the population references (****eTable 13****). The corresponding average HR was 5.92 (5.61-6.24) after restricting the analysis to individuals with a Charlson comorbidity score of zero, 6.15 (5.81-6.51) in individuals without an earlier healthcare visit, 5.37 (5.04-5.73) in those without earlier record of GI diseases, 5.65 (5.36-5.97) in those without earlier record of endoscopy, 5.56 (5.29-5.86) in those without previous colectomy or proctocolectomy, and 3.06 (2.71-3.45) in those with index date in January 2006 or later and without prescription of IBD medications before index date. Of note, although the HRs decreased in magnitude over time in all sensitivity analyses, all HRs remained statistically significant during the entire follow-up. "*

6. What proportion of the referent cohort had a colonoscopy during follow-up compared with those in the previous normal biopsy group?

**Reply:** Thank you for your suggestion. The proportion of recorded colonoscopy during follow-up was 10.4% vs. 4.3% in the individuals with a GI biopsy of normal mucosa and their matched reference individuals, respectively. We have now added such information in Table 1 (on pages 15-16) and Results.

On page 14:

*"The exposed individuals tended to have more healthcare visits, comorbidities, GI diseases, and endoscopies (e.g., colonoscopy and sigmoidoscopy) prior to the index date, as well as more colonoscopy during the follow-up than the population references and their unexposed full siblings (****Table 1****)."*

7. Are the code definitions of IBD validated in this dataset? Could you expand on their sensitivity and specificity. Also do these improve in more recent era, as is seen in many datasets like this - which could affect your results. What are the chances of them not picking up a diagnosis of IBD (in which case some patients may have already been diagnosed with IBD before the index biopsy and then only got coded after this)?

**Reply:** The positive predictive value (PPV) of our IBD definition has been validated before (PPV=95% (95%CI: 89%-99%) (clarified in the Methods). However, we have no data on the sensitivity and specificity of our IBD definition.

Although we have not evaluated the prevalence of IBD according to our definition, our earlier incidence data on IBD (**Ref 1**) are consistent with data from Busch et al. on the prevalence of IBD in Sweden, where the disease was diagnosed using 2 relevant ICD codes (**Ref 2**). We therefore speculate that the sensitivity and specificity of IBD definition should not be low. However, we have now acknowledged in the Discussion that while our IBD definition has a high PPV, its sensitivity and specificity has not been estimated.

Methods, on page 10:

*"We identified newly diagnosed IBD as having one relevant International Classification of Disease (ICD) code for IBD in the National Patient Register (available from 1964 onward), and one biopsy record indicating IBD in the ESPRESSO (see* ***eTable 1*** *for the ICD codes and histopathology SNOMED codes). A validation study has suggested a PPV of 95% (95%CI: 89%-99%) for this diagnostic approach[32]."*

Discussion, on page 28:

*"Fourth, while our IBD definition has a high PPV of 95%[32], its sensitivity and specificity have not been estimated."*

**Ref 1:** Forss A et al. A nationwide cohort study of the incidence of inflammatory bowel disease in Sweden from 1990 to 2014. Aliment Pharmacol Ther, 2021.

**Ref 2:** Busch K et al. Nationwide prevalence of inflammatory bowel disease in Sweden: a population-based register study[J]. Aliment Pharmacol Ther, 2014.

8. If your referent group was individually matched by birth year and calendar period then why is the median age at index date different between the cohorts? Presumably not all exposed individuals had the same number of matched referent individuals?

**Reply:** Thank you for this comment. This small difference (43.1 years for the exposed individual vs. 42.9 years for the reference individual) might be caused because they were matched by birth year, not exact age.

9. 32.2% of the exposed cohort had previously had an endoscopy. Can you specify if this was a gastroscopy, sigmoidoscopy, or colonoscopy? Had these patients not had a normal biopsy previously if the previous procedure was not counted as the index date?

**Reply:** We appreciate your comment. We have now reclassified these data into esophagogastroduodenoscopy, colonoscopy, and sigmoidoscopy and provided such information in the **Table 1** (see the below Table 3 adapted from **Table 1** in the manuscript).

Individuals with a previous endoscopy but without a GI histopathology report of normal mucosa were not considered as index persons in our study. Therefore the date of endoscopy was not considered as the index date. We have now clarified this in the Methods.

On page 9:

*"Date of biopsy for the exposed individuals and date of selection for population references or unexposed full siblings were used as the index date."*

| Table 3. Endoscopy before the index date among individuals with a lower GI biopsy of normal mucosa and their matched population references and unexposed full siblings | | | | | | |  | |
| --- | --- | --- | --- | --- | --- | --- | --- | --- |
|  | Population matched cohort, No. (%) | | *P* |  | Sibling cohort, No. (%) | | | *P* |
| Characteristics ^a^ | Normal mucosa (n=200,495) | References (n=989,484) |  |  | Normal mucosa (n=121,287) | Unexposed full siblings (n=221,179) | |  |
| Endoscopy | 64475 (32.2) | 25433 (2.6) | <0.001 |  | 40321 (33.2) | 11687 (5.3) | | <0.001 |
| EGD | 26710 (13.3) | 23095 (2.3) | <0.001 |  | 15911 (13.1) | 9254 (4.2) | | <0.001 |
| Colonoscopy | 51628 (25.8) | 6111 (0.6) | <0.001 |  | 33018 (27.2) | 4205 (1.9) | | <0.001 |
| Sigmoidoscopy | 7730 (3.9) | 1671 (0.2) | <0.001 |  | 4757 (3.9) | 1064 (0.5) | | <0.001 |
| EGD, esophagogastroduodenoscopy. | | | | | | |  | |
| ^a^ One individual may have multiple records of endoscopy before the index date. | | | | | | |  | |

10. I note the definition of endoscopy includes colonoscopy and sigmoidoscopy - this should be split from OGD so the reader has a better understanding of the data. I think a sensitivity analysis, where all individuals with previous lower GI endoscopy are excluded, is necessary.

**Reply:** Please see our response to the comment above.

We have also conducted a sensitivity analysis by excluding those with ***previous lower GI endoscopy*** from the analysis, and found the results (see below Table 4) were very similar to results from one of our sensitivity analyses where we excluded those with ***any previous endoscopy*** from the analysis (reported in **eTable 13**). We therefore chose not to present such new result in our manuscript.

| Table 4. Sensitivity analyses of IBD during follow-up in individuals with a lower GI biopsy result of normal mucosa, compared with their matched population references | | | | | | | |
| --- | --- | --- | --- | --- | --- | --- | --- |
| Subgroup | No. of events | | Average HR (95%CI) |  | Years since cohort entry, HR (95%CI) | | |
|  | Normal mucosa | References |  |  | 1 y | 10 y | 20 y |
| Excluding those with **any lower GI endoscopy** | 3821 | 4010 | 5.50 (5.21-5.81) |  | 7.05 (6.35-7.83) | 4.40 (4.12-4.70) | 3.00 (2.74-3.28) |
| Excluding those with **any endoscopy** | 3777 | 3976 | 5.65 (5.36-5.97) |  | 7.40 (6.67-8.22) | 4.46 (4.17-4.76) | 3.03 (2.77-3.31) |
| Hazard ratio was estimated from the flexible parametric survival model. All models were conditioned on matching set (birth year, sex, county of residence, and calendar period) and further adjusted for country of birth, educational attainment, number of healthcare visits, Charlson comorbidity index, and history of GI diseases. | | | | | | | |

11. Why are patients with previous colectomy not excluded? Presumably the colon was removed for a reason and would not have been normal at that time so it could not be the index normal biopsy.

**Reply:** Thank you for your comment. We have conducted a sensitivity analysis by excluding those with colectomy or proctocolectomy from the analysis and found similar result (reported in **eTable 13**) compared to the main result. We have now clarified this in the Methods and Results.

Methods, on page 13:

*"A number of sensitivity analyses were performed to assess the robustness of our results to potential influence from comorbidity and healthcare utilization.* *We restricted these analyses to: (a). individuals with a Charlson comorbidity index of zero; (b). individuals without a healthcare visit between 2 years and 6 months before the index date; (c). individuals free of GI diseases before the index date; (d). individuals free of previous endoscopy (see* ***eTable 2*** *for relevant codes); and (e).* ***individuals free of previous colectomy or proctocolectomy*** *(see* ***eTable 2*** *for relevant codes)."*

Results, on page 23:

*"The sensitivity analyses showed consistently higher risk of overall IBD among individuals with a normal lower GI mucosa, compared with the population references (****eTable 13****). The corresponding average HR was 5.92 (5.61-6.24) after restricting the analysis to individuals with a Charlson comorbidity score of zero, 6.15 (5.81-6.51) in individuals without an earlier healthcare visit, 5.37 (5.04-5.73) in those without earlier record of GI diseases, 5.65 (5.36-5.97) in those without earlier record of endoscopy,* ***5.56 (5.29-5.86) in those without previous colectomy or proctocolectomy****, and 3.06 (2.71-3.45) in those with index date in January 2006 or later and without prescription of IBD medications before index date. Of note, although the HRs decreased in magnitude over time in all sensitivity analyses, all HRs remained statistically significant during the entire follow-up."*

12. "the HR for overall IBD was higher in male (average HR=6.24 (5.77-6.76)) than female (5.11(4.78-5.46))" This comparison of Hazard Ratios is confusing to me. A ratio of ratios? I think it is better to just state the Hazard Ratios as they are without comparing them. It doesn't really mean anything to compare relative measures between them as there is no cross-over of the referent or exposed groups, they are distinct populations.

**Reply:** We appreciate your comment. We think understanding the difference across subgroups is important because it helps identify subgroups that have higher risk of developing outcomes. Moreover, as reviewer 2 suggested us to do more analyses to test whether the difference between subgroups is statistically significant, we therefore prefer to keep it as it is.

13. "in those without easier record of endoscopy" Typo - "earlier" I'm guessing?

**Reply:** Many thanks. We have now corrected the typo (on page 23).

14. "We observed that the risk increase for IBD was highest shortly after cohort entry, suggesting potential diagnostic delay." Can the authors comment on how this could have happened? Incomplete index colonoscopy but biopsies taken from normal area? Poor bowel preparation? Do they have data on these? Trainee endoscopist?

**Reply:** Thank you for your comment. We have now discussed it further in the Discussion. However, we lacked data on incomplete endoscopy, poor bowel preparation, and trainee endoscopists in our dataset.

On page 25:

*"The rapid initial risk increase for IBD after cohort entry might be driven by detection bias, surveillance bias, and reverse causation (e.g., the missed IBD diagnosis at time of biopsy with normal mucosa due to incomplete endoscopies or inadequate bowel preparation)."*

15. "Compared with UC (average HR=5.20), the risk increase was greater for CD (average HR=6.99); however, the cumulative incidence was lower for CD." This illustrates my earlier point that comparison of relative measures is not particularly helpful and the paper may represent the data better by focusing more on absolute measures like cumulative incidence. As a patient or clinician I am much more interested in the cumulative incidence because that tells me after a normal biopsy there is a 2.4% chance it is worth reinvestigating for IBD. The comparison to the general population is of academic interest but little clinical relevance. That said I found the HRs as a function of time in figure 1 interesting and useful to understand when these IBD diagnoses are more likely to occur.

**Reply:** We agree with your comment and have now edited the manuscript thoroughly to report more absolute risk estimates.

16. Is "preclinical" the right word? To me preclinical implies a period where there is active disease but symptoms have not yet developed. What the results suggest is actually the opposite - a period of being symptomatic before any histological change is detectable. I think this is really interesting but needs a different word. Perhaps "substantial symptomatic period before IBD is detectable with current histological methods"? I am sure you will find a more eloquent way of expressing this.

**Reply:** We thank you for such great comment and have now replaced "preclinical" with "symptomatic" throughout the manuscript accordingly.
