# Supplementary material for: Long-term risk of inflammatory bowel disease after endoscopic biopsy with normal mucosa: A population-based, sibling-controlled cohort study in Sweden
Source: PLoS Med. 2023 Feb 23;20(2):e1004185. doi: 10.1371/journal.pmed.1004185 (PMC9949679; doi:10.1371/journal.pmed.1004185)
Supplement: Attachment — Submitted filename: R2_letter to editor and reviewers_final.docx [file pmed.1004185.s005.docx]

**Requests from Editors:**

Please include the setting (Sweden) in the title.

**Reply:** The setting has now been added to the title.

New title:

*Long-term risk of inflammatory bowel disease after endoscopic biopsy with normal mucosa: A population-based, sibling-controlled cohort study* ***in Sweden***

Please include headline numbers (sample size and key findings) in your Author Summary. Please add an additional bullet under the second question in the Author Summary briefly describing the basic study design.

**Reply:** We appreciate your suggestions and have now added the headline numbers (sample size and key findings) and study design in the second question in the Author Summary.

On page 6 (page number in the version with track changes):

*"****What did the researchers find?***

- *In this population-based, sibling-controlled cohort study, we identified individuals with a lower (n=200,495) or upper (n=257,192) GI biopsy of normal mucosa, their individually matched general population references (n=989,484 and 1,268,897, respectively), and unexposed full siblings (n=221,179 and 274,529, respectively).*
- *Compared with population references and unexposed full siblings, individuals with a lower GI biopsy of normal mucosa had a persistently higher risk of overall IBD (average HR=5.56 and 3.27, respectively), ulcerative colitis (UC, average HR=5.20 and 3.27, respectively), and Crohn’s disease (CD, average HR=6.99 and 3.77, respectively).*
- *Individuals with an upper GI biopsy of normal mucosa were also at an increased risk of CD (average HR=2.93 and 2.39, respectively).*
- *The elevated risk of IBD persisted at least 30 years after a biopsy with normal mucosa."*

Please add an additional bullet under the first question in the Author Summary outlining the study question.

**Reply:** We have now outlined the study question in the Author Summary.

On page 6:

*"****Why was this study done?***

- *As the most frequent gastrointestinal (GI) histologic finding on endoscopy, a negative endoscopic result (i.e., normal mucosa) has been associated with a persistently decreased risk of colorectal cancer for up to 10 years. However, concerns have been raised about other long-term health outcomes among these individuals.*
- *We aimed to explore the long-term risk of inflammatory bowel disease (IBD) after an endoscopic biopsy with normal mucosa."*

Please label any items cited in the Supporting Information according to our guidelines.

**Reply:** We have now relabeled the cited items in the supporting information thoroughly according to the guidelines.

**Comments from Reviewers:**

**Reviewer #1:**

The response to the reviewer helps to better understand the matching process, but it's still not clear to me if birth year, sex, county of residence, and calendar period had equal weight for the matching. The authors have addressed all my other concerns appropriately.

**Reply:** Thank you for your further comment on the matching process. In the present study, since the exposed individual and reference individuals were individually matched by birth year, sex, county of residence, and calendar period, these four variables had equal weight in the matching process.

**Reviewer #2: Alex McConnachie, Statistical Review**

I thank the authors for their consideration of my original comments.

Regarding my comment about the need for a 6-month blanking period, the authors do not really answer this point. The HR curves over time all show a similar pattern, with highest risks early on, but a residual increased risk for up to 30 years. Would including events in the first 6 months alter this pattern at all? Or would you just see a continuation of the current trend? Perhaps all that would happen is that you would capture and measure the very high risk in those first 6 months.

**Reply:** Thank you for your comment. Starting follow-up immediately (i.e., including events in the first 6 months), from 6 months or 1 year (the cut-off point that you mentioned in the below comment) after biopsy could **lead to different average HRs**. Because only those who reached 6 months (or 1 year) after biopsy and free of the IBD can be enrolled in the latter two analyses. Excluding them probably results in an artificially lower proportion of susceptible individuals in the biopsy group, leading to different proportions of susceptible individuals in the biopsy and reference group (i.e., built-in selection bias [**Ref 1**]). However, as you mentioned in the below comment, flexible parametric survival model allows the HR to vary over time, therefore starting follow-up immediately, from 6 months or 1 year after biopsy would not greatly alter the HR pattern, especially the pattern in the later follow-up period (see the following three figures). Because the HR pattern in the later follow-up period was less likely to be affected by detection bias, surveillance bias, and reverse causation, which mainly play a role immediately after the cohort entry and derive the highest risk early on.

In the present study, we not only calculated average HR, but also reported the temporal pattern of HR over the entire follow-up period and presented specific HRs at 6 months, 1 year, 5 years, 10 years, 20 years, and 30 years after biopsy. We think that this way (i.e., reporting both average HR and the temporal pattern of HR) will leave the readership with a great freedom to understand the association between a GI biopsy of normal mucosa and risk of IBD.

Starting follow-up from 6 months after biopsy was *a priori* determined in our study proposal and comparable with previous studies [**Ref 2-4**].

Figure 1. Hazard ratio (HR) and 95% confidence interval (CI) of inflammatory bowel disease (IBD), comparing individuals with a GI biopsy result of normal mucosa with their matched population references. **Date of cohort entry was defined as the index date** (Index date was defined as the date of biopsy for the exposed individuals and date of selection for population references or unexposed full siblings)**.**

Figure 2. Hazard ratio (HR) and 95% confidence interval (CI) of inflammatory bowel disease (IBD), comparing individuals with a GI biopsy result of normal mucosa with their matched population references. **Date of cohort entry was defined as six months after the index date.**

Figure 3. Hazard ratio (HR) and 95% confidence interval (CI) of inflammatory bowel disease (IBD), comparing individuals with a GI biopsy result of normal mucosa with their matched population references. **Date of cohort entry was defined as one year after the index date.**

**Ref 1:** Hernan M A. The hazards of hazard ratios[J]. Epidemiology, 2010, 21(1): 13-5

**Ref 2:** Song M, Emilsson L, Hultcrantz R, et al. Long-Term Incidence and Mortality of Colorectal Cancer After Endoscopic Biopsy With Normal Mucosa: A Swedish-Matched Cohort Study[J]. Am J Gastroenterol, 2021, 116(2): 382-390

**Ref 3:** Lee J K, Jensen C D, Levin T R, et al. Long-term Risk of Colorectal Cancer and Related Deaths After a Colonoscopy With Normal Findings[J]. JAMA Intern Med, 2019, 179(2): 153-160

**Ref 4:** Pilonis N D, Bugajski M, Wieszczy P, et al. Long-Term Colorectal Cancer Incidence and Mortality After a Single Negative Screening Colonoscopy[J]. Ann Intern Med, 2020, 173(2): 81-91. doi:10.7326/M19-2477

That said, would the most natural explanation for the high early risk that is currently seen, be one of reverse causation? I.e. some of these people actually had disease at the initial biopsy, but this was not diagnosed. You could extend the blanking period to a year, to try to avoid this, but ultimately, where do you draw the line?

**Reply:** Please see our response to your first comment above.

We agree with the reviewer that the observed rapid initial increased IBD risk after cohort entry might be caused by reverse causation, detection bias, and surveillance bias. We have discussed it in the Discussion.

On page 26:

*"The rapid initial risk increase for IBD after cohort entry might be driven by detection bias, surveillance bias, and reverse causation (e.g., the missed IBD diagnosis at time of biopsy with normal mucosa due to incomplete endoscopies or inadequate bowel preparation)."*

However, given the type of model used, which allows the HR to vary over time, would you end up just "chopping off" the left-hand end of the curves in Figure 1? So, in the end, it makes no difference to the HR estimates in the long term, whether or not you include events early on.

**Reply:** We appreciate your comment, and please see our response to your first comment above.

What you could say is that the HR>1 early on is more likely to be due to some sort of reverse causality or other bias, whereas the HR>1 in later periods is less likely to be affected in this way. It is notable that the main conclusion of the paper is the IBD may have a lengthy period where patients have symptoms, before a successful diagnosis is made. In other words, the association between having a biopsy with normal mucosa and later diagnosis is due to reverse causation. I.e. patients in the early stages of the disease who have symptoms, may not be detected at biopsy, and it can take many years before a diagnosis is made.

**Reply:** Although detection bias, surveillance bias, and reverse causation might explain the observed rapid initial increased IBD risk after cohort entry, we think that these biases and reverse causation might be unlikely to fully explain the association between normal mucosa and ***later*** IBD diagnosis, because the association remained statistically significant during the entire 30 years of follow-up. We have discussed it in the Discussion.

On page 26:

*"The rapid initial risk increase for IBD after cohort entry might be driven by detection bias, surveillance bias, and reverse causation (e.g., the missed IBD diagnosis at time of biopsy with normal mucosa due to incomplete endoscopies or inadequate bowel preparation)."*

On page 28:

*"Normal mucosa might be a surrogate of a diverse range of GI symptoms or diseases before biopsy, which may be linked to IBD. Indeed, we found that individuals with normal mucosa were more likely to have a diagnosis of functional GI diseases (e.g., irritable bowel syndrome or functional dyspepsia) five years before biopsy in a previous study[22]. This is however unlikely to explain our results, as we noted a constantly positive association between normal mucosa and IBD during the entire 30 years of follow-up. Moreover, such concern might be partly alleviated by adjusting for Charlson comorbidity index, number of healthcare visit, and history of GI diseases in the analyses and by the similar results obtained after excluding individuals with comorbidity or GI diseases before GI biopsy."*

On other matters, I still feel that the paper tends to phrase things badly, in terms of implying that having normal mucosa puts patients at increased risk. For example, line 156 says "individuals with normal mucosa had an elevated subsequent risk"; line 221 "the effect of normal mucosa"; line 256 "association between normal mucosa and IBD"; Table 2 (and Table 3) footnote "the effect of normal mucosa"; line 372-3 "higher risk of overall IBD among individuals with a normal lower GI mucosa"; line 384 "HR of 2.15 for normal lower GI mucosa"… I am sure there are other similar instances.

**Reply:** We appreciate your suggestions and have now revised the manuscript accordingly.

My issue is that it is improbable that having normal mucosa is associated with outcomes. I presume that the vast majority of the unexposed groups also had normal mucosa. The difference is that the exposed group had symptoms, sufficient to warrant undergoing a biopsy.

**Reply:** We agree with the reviewer's opinion and have discussed it in the Discussion.

On page 28:

*"Normal mucosa might be a surrogate of a diverse range of GI symptoms or diseases before biopsy, which may be linked to IBD. Indeed, we found that individuals with normal mucosa were more likely to have a diagnosis of functional GI diseases (e.g., irritable bowel syndrome or functional dyspepsia) five years before biopsy in a previous study[22]. "*

Otherwise, I am happy with the authors' responses to my original comments. On rereading the paper, one or two other things came up.

Table 1 now includes p-values, many of which are highly statistically significant, due to the vary large sample size. The differences between groups are not necessarily vary large (e.g. for age, comparing the exposed group with the general population, the difference is 0.2 years, but the p-value is <0.001), and may not be clinically relevant. Is there any other way to compare the groups? Some sort of standardized mean difference, perhaps?

**Reply:** Thank you for bringing this to our attention. As suggested by the reviewer, we have now used the standardized difference to examine the balance of a covariate between the exposed and unexposed groups, added this method in the Methods, and revised Table 1 (on pages 15-16) and Table L in S1 Appendix (similar to Table 1, but showing the characteristics of individuals with an **upper** GI biopsy of normal mucosa and their matched population references and unexposed full siblings).

Methods, on page 11:

*"Standardized difference was used to examine the balance of a covariate between the exposed and unexposed groups and imbalance was defined as a standardized difference value greater than 0.2[39]."*

Were the exposed group more likely to undergo biopsies after the index procedure? How many did they have? Is it possible that if you have enough biopsies, you will get a diagnosis of something eventually?

**Reply:** Thank you for the comment. The exposed group were more likely to undergo biopsy during the follow-up time. The percentage was 24.7% vs. 11.9% for those who had a **lower** GI biopsy of normal mucosa and their matched population references and 26.5% vs. 13.2% for those who had an **upper** GI biopsy of normal mucosa and their matched population references. The median number of follow-up biopsies were around 2.5. Exploring the following diagnosis of individuals with a biopsy of normal mucosa is interesting, we however think it is not within the scope of this paper.

Line 385-6 says "an unmeasured confounder that confers a 3.72-fold increase in normal lower GI mucosa". Does this make sense? How can you have a confounder that "causes" normal mucosa?

**Reply:** Thank you for the comment. The value 3.72 is an E-value, which is a theoretical value. In present study, we calculated the E-value to identify the minimum strength of the association an unmeasured confounder would need to have with both exposure and outcome to explain away the observed association between normal mucosa and IBD.

Although it seems there is no confounder that could influence the risk of undergoing GI biopsy examination with a biopsy result of normal mucosa in observational studies, we, for example, indeed observed a strong association between a history of irritable bowel syndrome and risk of a biopsy result of normal mucosa (odds ratio>10) in our previous study **[Ref 1]**. Patients with irritable bowel syndrome were also more likely to have an increased risk of IBD (adjusted rate ratio>8) **[Ref 2]**. Therefore we speculate that normal mucosa might be a surrogate of a diverse range of GI symptoms or diseases before biopsy, which may be linked to IBD. We have discussed it in the Discussion.

On page 28:

*"Our study also has limitations. First, lack of data on indications for biopsy is a concern. Normal mucosa might be a surrogate of a diverse range of GI symptoms or diseases before biopsy, which may be linked to IBD. Indeed, we found that individuals with normal mucosa were more likely to have a diagnosis of functional GI diseases (e.g., irritable bowel syndrome or functional dyspepsia) five years before biopsy in a previous study[22]."*

**Ref 1:** Sun J, Ludvigsson J F, et al. Gastrointestinal biopsies and amyotrophic lateral sclerosis - results from a cohort study of 1.1 million individuals[J]. Amyotroph Lateral Scler Frontotemporal Degener, 2021, 22(5-6): 410-418.

**Ref 2:** Porter, Chad K., et al. Risk of inflammatory bowel disease following a diagnosis of irritable bowel syndrome. BMC gastroenterology 12.1 (2012): 1-10.

Line 433-4 says "Both normal mucosa and IBD were objectively ascertained through validated methods with high PPVs (both ≥95%)." This only gives the PPV. What about the NPV? Is it known, of those with a result of normal mucosa, how many would be expected to have IBD? To what extent does this explain the observed associations?

**Reply:** Thank you for the comment. However, we did not have such data to calculate the negative predictive value. This would require us to examine patient charts in a large number of individuals with normal mucosa (i.e. negative for IBD), but we did not have access to such patient charts.

**Reviewer #3:**

Thank you for addressing my comments. Congratulations on a very interesting paper. I have no further comments.

**Reply:** Thank you for your positive feedback.
